# The glycosyltransferase UGT76B1 modulates *N*-hydroxy-pipecolic acid homeostasis and plant immunity

**DOI:** 10.1093/plcell/koaa045

**Published:** 2021-01-11

**Authors:** Lennart Mohnike, Dmitrij Rekhter, Weijie Huang, Kirstin Feussner, Hainan Tian, Cornelia Herrfurth, Yuelin Zhang, Ivo Feussner

**Affiliations:** 1 Department of Plant Biochemistry, Albrecht-von-Haller-Institute for Plant Sciences, University of Goettingen, D-37077 Goettingen, Germany; 2 Department of Botany, University of British Columbia, Vancouver, BC V6T 1Z4, Canada; 3 Service Unit for Metabolomics and Lipidomics, Goettingen center for Molecular Biosciences (GZMB), University of Goettingen, D-37077 Goettingen, Germany; 4 Department of Plant Biochemistry, Goettingen Center for Molecular Biosciences (GZMB), University of Goettingen, D-37077 Goettingen, Germany

## Abstract

The tradeoff between growth and defense is a critical aspect of plant immunity. Therefore, the plant immune response needs to be tightly regulated. Salicylic acid (SA) is an important plant hormone regulating defense against biotrophic pathogens. Recently, *N*-hydroxy-pipecolic acid (NHP) was identified as another regulator for plant innate immunity and systemic acquired resistance (SAR). Although the biosynthetic pathway leading to NHP formation is already been identified, how NHP is further metabolized is unclear. Here, we present UGT76B1 as a uridine diphosphate-dependent glycosyltransferase (UGT) that modifies NHP by catalyzing the formation of 1-*O*-glucosyl-pipecolic acid in *Arabidopsis thaliana*. Analysis of T-DNA and clustered regularly interspaced short palindromic repeats (CRISPR) knock-out mutant lines of *UGT76B1* by targeted and nontargeted ultra-high performance liquid chromatography coupled to high-resolution mass spectrometry (UHPLC-HRMS) underlined NHP and SA as endogenous substrates of this enzyme in response to *Pseudomonas* infection and UV treatment. *ugt76b1* mutant plants have a dwarf phenotype and constitutive defense response which can be suppressed by loss of function of the NHP biosynthetic enzyme FLAVIN-DEPENDENT MONOOXYGENASE 1 (FMO1). This suggests that elevated accumulation of NHP contributes to the enhanced disease resistance in *ugt76b1*. Externally applied NHP can move to distal tissue in *ugt76b1* mutant plants. Although glycosylation is not required for the long-distance movement of NHP during SAR, it is crucial to balance growth and defense.

## Teaser

The UDP-dependent glycosyltransferase 76B1 influences the homeostasis of *N*-hydroxy-pipecolic acid in *Arabidopsis thaliana* by catalyzing the formation of its glycoside form.

## Introduction

Plants are constantly exposed to biotic and abiotic stress. To deal with external threats, plants have developed an impressive repertoire of chemical compounds. However, there is a trade-off between defense and growth as shown in autoimmune mutants such as *snc2*-1D, *npr1*-1, and *s3h s5h*, which accumulate high levels of defense hormones and exhibit severe dwarf phenotypes (Zhang et al., 2010, 2017). To balance growth and defense responses, plants constantly monitor and adjust the homeostasis of these compounds. Dynamic changes of the levels of immune signaling molecules allow plants to react rapidly and appropriately to danger (Hartmann and Zeier, 2019; Huang et al., 2020). The biosynthesis, transport, and homeostasis of the signaling molecules are therefore strictly regulated to prevent unintended consequences.

Two signaling molecules, salicylic acid (SA) and *N*-hydroxy-pipecolic acid (NHP), are particularly important in plant defense against biotrophic pathogens. Together they orchestrate the immune response in the local tissue to prevent pathogen spread (Hartmann et al., 2018; Guerra et al., 2020). Locally produced defense signals are further translocated to distal parts of the plant, leading to massive transcriptional, and metabolic reprogramming in the naive tissues, which enables a quick and robust response to subsequent infections (Bernsdorff et al., 2016). This induced immunity in distal tissue is termed as systemic acquired resistance (SAR). Most of the signaling molecules participating in the induction of SAR can be found in the phloem upon infection (Fu and Dong, 2013). The effect of SA and NHP in the context of plant immunity has been well documented (Chen et al., 2018; Hartmann et al., 2018; Zhang and Li, 2019; Huang et al., 2020).

Biosynthesis of SA is divided into two major routes that result in SA formation in planta: The phenylpropanoid or PHENYLAMMONIA LYASE (PAL) pathway and the ISOCHORISMIC ACID SYNTHASE 1 (ICS1) pathway (Yalpani et al., 1993; Wildermuth et al., 2001). Nevertheless, in *Arabidopsis thaliana* ∼90% of endogenous SA derives from chloroplast-derived isochorismic acid, which is exported to the cytosol via ENHANCED DISEASE SUSCEPTIBILITY 5 (EDS5) and conjugated to glutamate by AvrPphB SUSCEPTIBLE 3 (PBS3). The formed isochorismic acid-9-glutamic acid then spontaneously decomposes into SA and enolpyruvyl-*N*-glutamic acid (Rekhter et al., 2019b). Furthermore, ENHANCED PSEUDOMONAS SUSCEPTIBILITY 1 (EPS1) has been shown to enhance SA formation from isochorismic acid-9-glutamic acid (Torrens-Spence et al., 2019).

**Figure 1:**
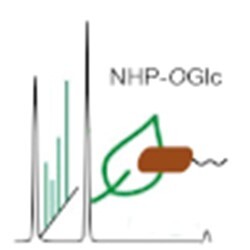
Biosynthesis of NHP-*O*Glc. The biosynthesis of NHP-*O*Glc starts from L-lysine, which is converted by ALD1 to ε-amino-α-keto caproic acid (Song et al., 2004; Navarova et al., 2012; Vogel-Adghough et al., 2013). The compound spontaneously cyclizes to P2C and is reduced by SARD4 to Pip (Ding et al., 2016; Hartmann et al., 2017). FMO1 hydroxylates Pip to form NHP, the biologically active pipecolate (Chen et al., 2018; Hartmann et al., 2018). In a last step, NHP is glucosylated at the hydroxyl functional group to form NHP-*O*Glc.

NHP was recently discovered as a signaling compound for plant defense against biotrophic pathogens (Chen et al., 2018; Hartmann et al., 2018). So far, research has focused on the biosynthesis of NHP from lysine. In the first step, the α-aminotransferase AGD2-LIKE DEFENSE RESPONSE PROTEIN 1 (ALD1) catalyzes the transamination of lysine into ε-amino-α-keto caproic acid (Song et al., 2004; Navarova et al., 2012; Vogel-Adghough et al., 2013) (Figure 1). This compound spontaneously cyclizes and thereby yields Δ^1^-piperideine-2-carboxylic acid (P2C). In a second step, the ketimine reductase SAR-DEFICIENT 4 (SARD4) catalyzes the formation of pipecolic acid (Pip) from P2C (Ding et al., 2016; Hartmann et al., 2017). Pip requires *N*-hydroxylation to NHP in order to reach its full protective capacity. This activation is catalyzed by FLAVIN-DEPENDENT MONOOXYGENASE 1 (FMO1) (Chen et al., 2018; Hartmann et al., 2018) (Figure 1).

One important strategy to maintain a preferred concentration of an active metabolite is chemical modification, which can change the bioavailability and activity of the compound. Different modifications of SA such as hydroxylation and methylation have been described (Song et al., 2009; Zhang et al., 2017). SA itself as well as its catabolites can be further xylosylated (addition of the pentose xylose) and glycosylated (addition of a hexose) (Song et al., 2008; Bartsch et al., 2010; Huang et al., 2018). The transfer of an activated sugar moiety onto a target molecule is predominantly catalyzed by the widespread enzyme family of uridine diphosphate (UDP)-DEPENDENT GLYCOSYL TRANSFERASES (UGTs). The closely related UGT74F1 and UGT74F2 catalyze the formation SA-glycoside (SAG) and SA glucose ester (SGE), respectively (Dean and Delaney, 2008; George Thompson et al., 2017). Another enzyme UGT71C3 was recently shown to be responsible for the biosynthesis of methyl-SA glycoside (Chen et al., 2019). Despite the high abundance of these glycosides upon stress, the biological significance of the formation of these compounds is elusive. Blocking glycosylation of SA has been shown to result in enhanced disease resistance (Noutoshi et al., 2012). In tobacco (*Nicotiana tabacum*), SAG is transported from the cytosol into vacuoles, suggesting that the glucosides are a storage form of SA. On the other hand, the formation of SAG may be important for the vascular transport, as there is evidence that SAG can be hydrolyzed back into SA in the extracellular space (Hennig et al., 1993; Seo et al., 1995).

So far, only one metabolite of NHP was identified, namely NHP-*O*-glycoside (NHP-*O*Glc) (Chen et al., 2018; Hartmann et al., 2018). Intriguingly, externally supplied NHP can be found in distal tissues in uninfected *fmo1* mutant plants as NHP and NHP-*O*Glc, suggesting that at least one of these molecules is mobile *in planta* (Chen et al., 2018). Until now, neither the function of NHP-*O*Glc nor the enzyme that catalyzes the glycosylation of NHP has been identified. Here we report that UGT76B1, which was previously reported to glycosylate SA and 2-hydroxy-3-methyl-pentanoic acid (isoleucic acid, ILA), catalyzes the formation of NHP-*O*Glc (von Saint Paul et al., 2011; Noutoshi et al., 2012; Maksym et al., 2018). UGT76B1 has strong in vitro activity toward NHP and no detectable amount of NHP-*O*Glc is synthesized in *ugt76b1* mutant plants, which results in increased NHP accumulation, a dwarf phenotype, and enhanced disease resistance against biotrophic pathogens. Moreover, we show that externally applied NHP is mobile to distal tissue in the absence of UGT76B1 and that transport of NHP seems not to depend on further glycosylation.

## Results

### Nontargeted metabolome analysis of infected leaf tissue revealed NHP as the in vivo substrate of UGT76B1

Searching for the protein that catalyzes the formation of NHP-*O*Glc, we identified *UGT76B1* as a recurring candidate gene from several studies (von Saint Paul et al., 2011; Noutoshi et al., 2012; Gruner et al., 2013; Hartmann et al., 2018). *UGT76B1* was classified as a SAR gene in these studies. In addition, by screening online co-expression databases, we established that *UGT76B1* is co-expressed with *FMO1*. This encouraged us to further investigate the role UGT76B1 in plant immunity. The loss-of-function mutant *ugt76b1-*1 showed enhanced resistance against *Pseudomonas* infections (von Saint Paul et al., 2011; Noutoshi et al., 2012; Maksym et al., 2018). Although UGT76B1 has previously been shown to exhibit SA glycosyltransferase activity, the enzyme has a high level of substrate promiscuity in vitro. Additional substrates are ILA, leucic acid, 2-ethyl-2-hydroxybutyric acid, and valic acid (von Saint Paul et al., 2011; Noutoshi et al., 2012; Maksym et al., 2018). Since UGT76B1 has been shown to influence SA metabolism, we wondered if UGT76B1 has other substrates in vivo.

**Figure 2:**
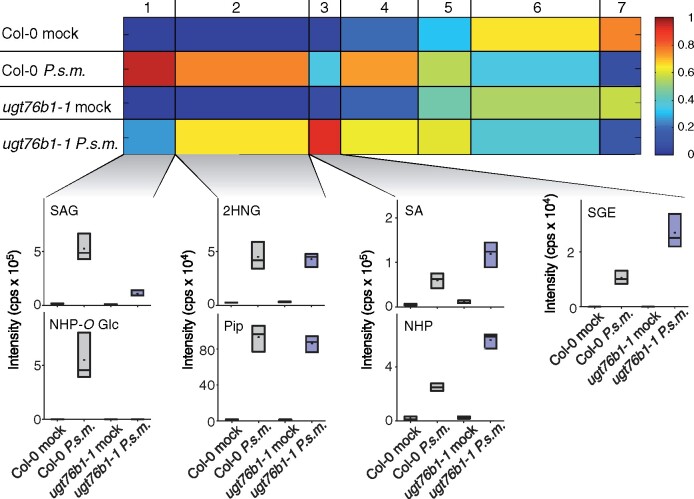
Nontargeted metabolomics revealed NHP as a substrate of UGT76B1 in vivo. Col-0 and *ugt76b1*-1 mutant plants were infiltrated with MgCl_2_ (mock) or *Pseudomonas syringae* ES4326 (*P.s.m.*) at OD_600_ = 0.05. Samples were collected 24 hpi. Metabolites of the polar extraction phase were analyzed by a metabolite fingerprinting approach based on UHPLC-HRMS. Intensity-based clustering by means of one-dimensional self-organizing maps of the relative intensities of 448 metabolite features (FDR < 0.005) in seven clusters is shown. The heat map colors represent average intensity values according to the color map on the right-hand side. The width of each cluster is proportional to the number of features assigned to this cluster. Box plots for selected metabolites of the indicated clusters are shown. Borders represent the high and low value of the measurement and horizontal lines represent the median value. The identity of the metabolites was unequivocally confirmed by UHPLC-HRMSMS analyses. Data represent *n* = 3 replicates. Each replicate represents an individual pool of 4–6 leaves of six plants per condition. The results were confirmed by a second independent experiment.

We conducted a nontargeted metabolome analysis on Col-0 and *ugt76b1*-1 leaves after mock or *Pseudomonas* treatment. The dataset obtained by the nontargeted ultra-high performance liquid chromatography coupled to high-resolution mass spectrometry (UHPLC-HRMS) analysis showed relative intensity profiles of 448 metabolite features [false discovery rate {FDR} < 0.005], which were arranged into seven clusters by means of one-dimensional self-organizing maps (Figure 2). NHP-*O*Glc was not detectable in infected *ugt76b1*-1 mutant plants and SAG was strongly reduced compared to the *Pseudomonas syringae* ES4326 (*P.s.m.*) infected wild-type plants (Col-0; Figure 2, Cluster 1). In contrast to that, NHP and SA showed a three- and two-fold accumulation, respectively, in infected *ugt76b1*-1 plants compared to the respective wild-type plants (Cluster 3). Interestingly, the NHP precursor Pip, as well as 2HNG as a fragment of the SA-precursor isochorismic acid-9-glutamic acid, showed comparable amounts in infected wild-type and *ugt76b1*-1 mutant plants (Cluster 2). We could not find evidence for additional substrates or products of UGT76B1 under our conditions with the nontargeted approach. However, we detected increased levels of the second SA-derived metabolite SGE in *ugt76b1*-1 plants after infection (Cluster 3). Together, the experiment leads to the identification of NHP as an in vivo substrate of UGT76B1.

### *UGT76B1* loss-of-function mutant plants do not accumulate NHP-*O*Glc

**Figure 3:**
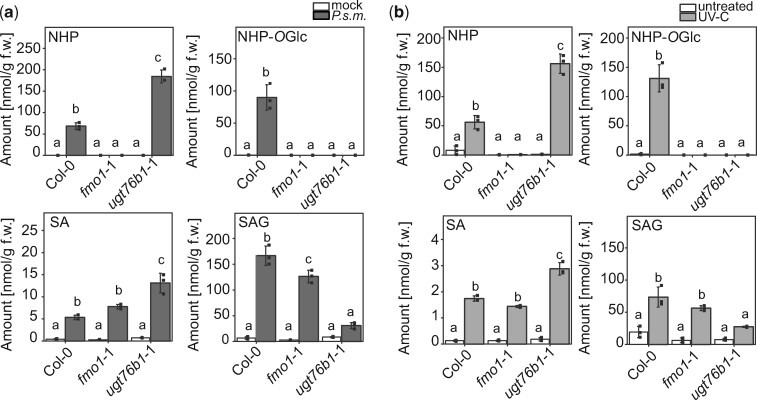
*UGT76B1* loss-of-function mutant plants are unable to synthesize NHP-*O*Glc. Absolute amounts of NHP, NHP-*O*Glc, SA, and SAG in wild-type (Col-0), *fmo1*-1, and *ugt76b1*-1 plants after infection with *P.s.m.* (A) or UV treatment (B). Three leaves of 6-week-old plants, grown under short-day conditions (8-h light period), were infiltrated with *P.s.m.* at OD_600_ = 0.05 in 10 mM MgCl_2_ (*P.s.m.*) or with 10 mM MgCl_2_ (mock). Twenty-four hpi, leaves were harvested and analyzed using UPLC-nanoESI-QTRAP-MS. Plants grown under long-day conditions (16-h light period) were treated for 20 min with UV-C. Twenty-four hours post-UV-C treatment, leaves were harvested and analyzed using quantitative UPLC-nanoESI-QTRAP-MS with authentic internal standards. Data represent the absolute amount of analyte in nmol/g fresh weight (f.w.). Error bars represent standard deviation. Letters indicate statistical differences (*P* < 0.05, one-way ANOVA; *n* = 3). Replicates represent individual pools of 4–6 leaves from six plants per condition. The results were confirmed by a second independent experiment.

In addition to nontargeted metabolome analysis, we quantitatively analyzed the amount of NHP, NHP-*O*Glc, SA, and SAG in wild-type (Col-0), *fmo1-*1, and *ugt76b1*-1 plants after infection with *P.s.m.* (Figure 3A). Twenty-four hours post-infection (hpi), wild-type plants accumulated NHP and NHP-*O*Glc to levels of 68 and 89 nmol/g fresh weight (f.w.), as well as of SA and SAG to 7 and 166 nmol/g f.w., respectively. *ugt76b1*-1 plants exhibited nearly a three-fold higher accumulation of NHP (184 nmol/g f.w.) compared to wild-type, whereas NHP-*O*Glc was not detected in the mutant after infection. As expected, *fmo1*-1 plants, which cannot generate NHP from Pip, accumulated neither NHP nor NHP-*O*Glc. Additionally, we observed an about 2.5-fold higher accumulation of SA after infection in *ugt76b1*-1 plants compared to the wild-type, whereas *fmo1*-1 plants exhibited comparable SA levels to the wild-type, and moderately reduced SAG levels.

Similar results were obtained when we used UV-C to stimulate the production of NHP and SA independently of pathogen infection (Yalpani et al., 1994; Rekhter et al., 2019a). Twenty-four hour post-UV-C-treatment, we detected 56 and 131 nmol/g f.w. of NHP and NHP-*O*Glc as well as 1.74 and 73 nmol/g f.w. of SA and SAG in wild-type plants (Figure 3B). In *fmo1*-1 plants, no detectable amounts of NHP and NHP-*O*Glc were found after UV-C treatment, while SA and SAG accumulated to wild-type levels. In *ugt76b1*-1 plants, we observed a nearly three-fold increase in NHP compared to wild-type plants, but no formation of NHP-*O*Glc was detectable. There is also an increase in SA accumulation (2.87 nmol/g f.w.) and a decrease in SAG accumulation (27 nmol/g f.w.) in *ugt76b1*-1. Together, these data strengthen the hypothesis that NHP-*O*Glc formation is dependent on a functional UGT76B1 enzyme, as was additionally confirmed with two independent deletion mutant alleles of *UGT76B1* (Supplemental Figure S1).

### UGT76B1 acts downstream of FMO1 thereby regulating plant immunity

**Figure 4:**
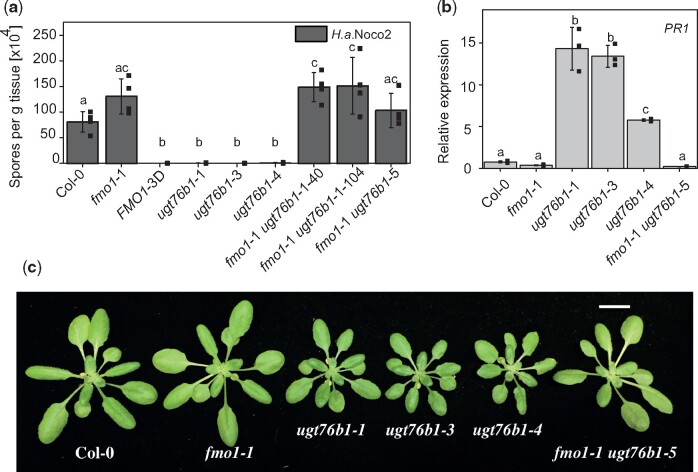
Rescue of *ugbt76b1* mutant phenotypes by introduction of the *fmo1-*1 mutation. A, Growth of *H. a*. Noco2 on wild-type (Col-0), *fmo1*-1, *FMO1*-3D, *ugt76b1*-1, *ugt76b1*-3, *ugt76b1*-4, *fmo1*-1 *ugt76b1*-40, *fmo1*-1 *ugt76b1*-104, and *fmo1*-1 *ugt76b1*-5 plants. Two-week-old seedlings were sprayed with *H.a.* Noco 2 spore suspension (5 × 10^4^ spores/mL). Infection was scored 7 days after infection. Error bars represent standard deviation. Letters indicate statistical differences (*P* < 0.05, one-way ANOVA; *n* = 4). Each replicate represents the spore count on a single plant. B, Basal *PR1* gene expression in 4-week-old plants of the indicated genotypes determined via quantitative RT-PCR. Error bars represent standard deviation. Letters indicate statistical differences (*P* < 0.05, one-way ANOVA; *n* = 3). Replicates represent a pool of 4–6 leaves per genotype. C, Growth phenotypes of Col-0, *fmo1*-1, *ugt76b1*-1, *ugt76b1*-3, *ugt76b1*-4, and *fmo1*-1 *ugt76b1*-5. Photographs are of 4-week-old plants grown under long-day conditions (16-h light/8-h dark cycle). Scale bar is 1 cm.

We hypothesized that increased NHP accumulation in *ugt76b1*-1 plants after infection is due to its impaired glycosylation and that the dwarfed and enhanced resistance phenotype requires NHP. Furthermore, we assumed that UGT76B1 acts downstream of FMO1. To test this hypothesis, we assessed the growth of *Hyaloperonospora arabidopsis* (*H*.*a*.) Noco 2 on Col-0, *fmo1-*1, *FMO1*-3D (a gain-of-function mutant for *FMO1*), three mutant alleles of *UGT76B1* (*ugt76b1*-1, -3, and -4) and three *fmo1-*1 *ugt76b1* double knock-out mutant lines (*fmo1-*1 *ugt76b1-*5, *fmo1*-1 *ugt76b1*-1-40, and *fmo1*-1 *ugt76b1*-1-104; Figure 4). In comparison to Col-0, *FMO1*-3D showed high resistance against *H*. *a*. Noco 2, while *fmo1*-1 was more susceptible. *ugt76b1*-1, -3, and -4 exhibited strong resistance, but the double mutant lines showed similar susceptibility as *fmo1*-1 (Figure 4A). Additionally, we found that basal *PR1* expression is enhanced in all three *ugt76b1* alleles compared to Col-0 (Figure 4B), consistent with findings from a previous report (von Saint Paul et al., 2011). In contrast, the expression level of *PR1* is similar in *fmo1*-1 *ugt76b1*-5 and *fmo1*-1. In addition, the dwarf phenotype and dark green leaf color in the *ugt76b1* alleles are suppressed in the *fmo1*-1 *ugt76b1*-5 double mutant (Figure 4C). The *fmo1*-1 *ugt76b1*-1 double mutant plants accumulate neither NHP nor NHP-*O*Glc (Supplemental Figure S2). Altogether, the data indicate that UGT76B1 acts downstream of FMO1 and that NHP is required for both the enhanced resistance and dwarf phenotype of *ugt76b1* plants.

### Increased accumulation of NHP in *ugt76b1* plants underlines the importance of turnover via UGT76B1

**Figure 5:**
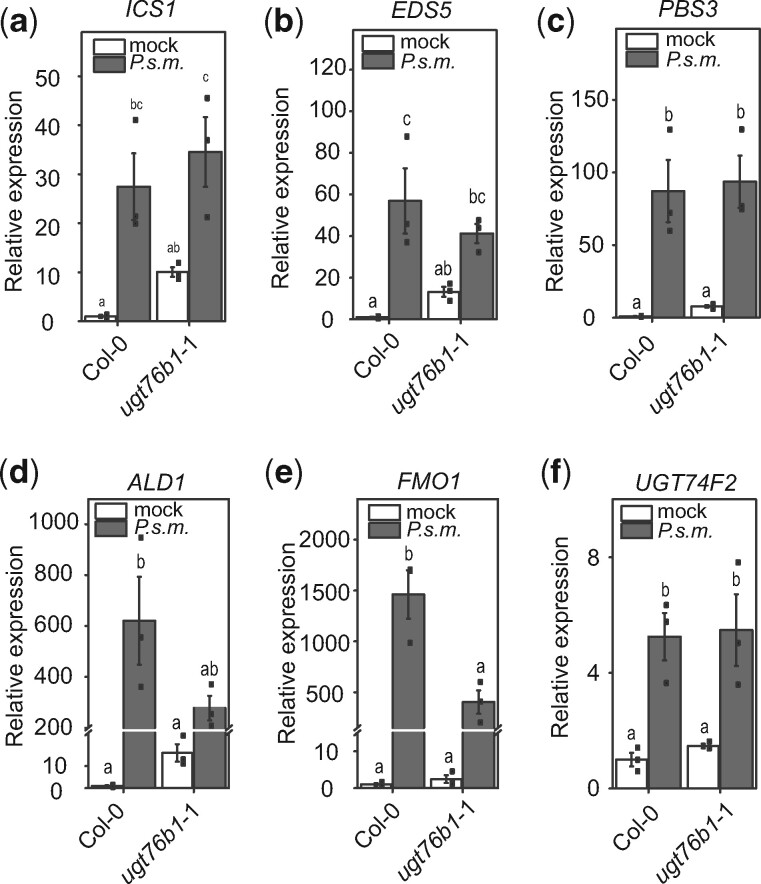
Comparisons between transcript levels of *ICS1*, *EDS5*, *PBS3*, *ALD1*, *FMO1*, and *UGT74F2* in *ugt76b1*, and the wild-type. Transcript abundance of genes encoding SA and NHP biosynthetic enzymes was analyzed in wild-type and *ugt76b1*-1 plants after infection with *P.s.m.*. Three leaves of 4–6-week-old plants were treated with *P.s.m.* (OD_600_ = 0.001). Leaves were harvested 24 hpi and analyzed via RT-PCR using cDNA generated by reverse-transcriptase reaction as templates. Error bars represent standard deviation. Letters indicate statistical differences (*P* < 0.05, one-way ANOVA; *n* = 3). Replicates represent pools of 4–6 leaves of six plants per condition. Graph d includes an axis break from 25 to 200. Graph e includes an axis brake from 15 to 150.

Next, we wondered whether the enhanced accumulation of NHP and SA in the *ugt76b1* mutants after infection is due to impaired turnover or increased biosynthesis of NHP and SA. As an indirect measure, we analyzed the transcript levels of SA and NHP biosynthetic genes 24 hpi with *P.s.m*. by quantitative real time-polymerase chain reaction (RT-PCR). The transcript abundance of the SA biosynthetic genes *ICS1*, *EDS5*, and *PBS3* (Figure 5, A–C) was similar in the wild-type and *ugt76b1-*1 mutant. Interestingly, transcripts of all three genes were upregulated in the mock-treated *ugt76b1-*1, suggesting that the basal expression levels of these SA biosynthetic genes are higher in the *UGT76B1* knock-out background. This notion is supported by the transcript levels of *PR1* and *PR2* after mock treatment (Supplemental Figure S3). Despite the increased amount of NHP (Figure 3A), the transcript levels of NHP-biosynthetic genes *ALD1* and *FMO1* are significantly reduced in *ugt76b1*-1 compared to the wild-type. As a control, we monitored the transcript level of *UGT74F2* in Col-0 and *ugt76b1*-1. The transcript abundance of *UGT74F2* did not change after infection in Col-0 and *ugt76b1*-1 plants (Figure 5). Taken together, the increased SA and NHP levels in *ugt76b1* mutants upon pathogen infection are unlikely caused by increased biosynthesis as shown on the level of transcription of the biosynthetic genes, since the respective transcripts are not higher in *ugt76b1*-1 than in the wild-type. These findings may support that UGT76B1 plays a central role in the turnover of NHP and influences the formation of SAG.

### UGT76B1 catalyzes the glycosylation of NHP in vitro

**Figure 6:**
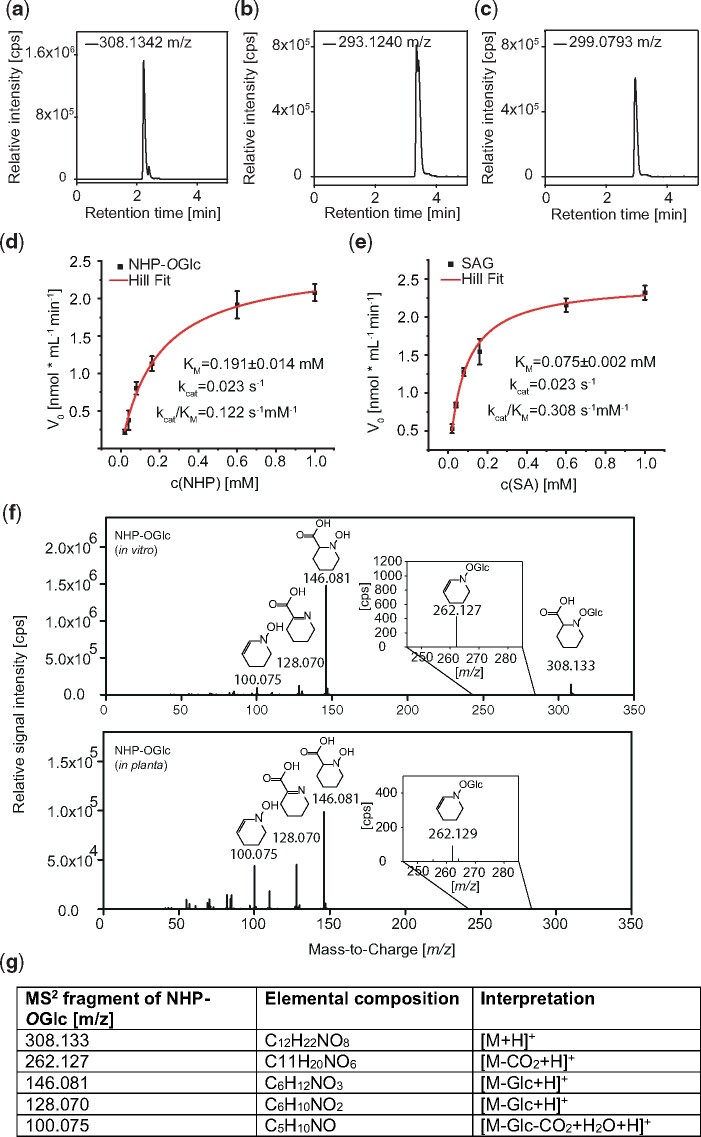
Glycosylation of SA, ILA, and NHP by UGT76B1 in vitro. Activity assays were carried out using NHP, ILA, and SA as substrates for the recombinant UGT76B1. Extracted ion chromatograms of the reaction products (A) NHP-*O*Glc (*m*/*z* 308.1342), (B) ILA-Glc (*m*/*z* 293.1240), and (C) SAG (*m*/*z* 299.0793) are shown. An aliquot of 10 µg of recombinant UGT76B1 was incubated with 50 µM substrate and 500 µM UDP-Glc at 30°C for 30 min. The reaction was stopped by adding 25% (*v/v*) acetonitrile. The kinetic constants (*K*_M_, *k*_cat_, and *k*_cat_/*K*_M_) of UGT76B1 were determined for the substrate NHP [coefficient of determination (*R*^2^ = 0.996) (D) and SA (*R*^2^ = 0.997) (E)], respectively. Mean signal area of the respective products (NHP-*O*Glc or SAG) after 5 min incubation at 25°C from three replicates at six different substrate concentrations. Non-linear Hill regression was performed with Origin Pro 2020 (OriginLab Corporation, Northampton, MA, USA). F, UHPLC-HRMS/MS-fragment spectra of enzymatically synthesized NHP-*O*Glc (in vitro) and of NHP-*O*Glc extracted from wild-type plants after infection with P.s.m. (*in planta*) is shown. The analytical fragment *m/z* 262.127 represents the NHP-*O*Glc molecule without the carboxy group, being specific for an *O*-glycosylation. G, Interpretation of the NHP-*O*Glc fragments using the accurate mass information and the deduced elemental composition. All samples were measured via UHPLC-HRMS-analysis. The results were confirmed by a second independent experiment.

In addition, we checked whether UGT76B1 can glycosylate NHP in vitro. The His-tagged UGT76B1 was heterologously expressed in *Escherichia coli* and purified to homogeneity by affinity chromatography and size exclusion chromatography (Supplemental Figure S4). The enzymatic reaction of recombinant UGT76B1 with NHP and UDP-glucose as substrates was monitored by UHPLC-HRMS. As shown in Figure 6A, UGT76B1 catalyzes in vitro formation of NHP-*O*Glc (*m*/*z* 308.1342, retention time [RT] 2.12 min). We also confirmed glycosylation of SA and ILA by UGT76B1 (von Saint Paul et al., 2011; Noutoshi et al., 2012). The formation of the respective glucosides SAG (*m*/*z* 299.0793, RT 3.14 min) and ILA-glycoside (ILA-Glc) (*m*/*z* 293.1240, RT 3.35 min) is shown in Figure 6, B and C. In addition, we determined the Michaelis–Menten constant (*K*_M_) for SA and NHP. We quantified the respective product signal area for NHP-*O*Glc and SAG via UPLC-nanoESI-QTRAP-MS, resulting in *K*_M_(NHP) = 191 ± 14 µM and *K*_M_(SA) = 75 ± 2 µM (Figure 6, D and E) and a catalytic efficiency (*k*_cat_/*K*_M_) of *k*_cat_/*K*_M_(NHP) = 0.122 s^−1^ mM^−1^ as well as *k*_cat_/*K*_M_(SA) = 0.308 s^−1^ mM^−1^. The data suggest that the glycosylation of SA by UGT76B1 is ∼2.5-fold more efficient than the glycosylation of NHP. We further investigated the site of glycosylation by mass spectrometry (MS)-fragmentation studies on enzymatically produced (in vitro) and *in planta* NHP-*O*Glc (Figure 6, F and G), which have identical RTs in our chromatographic separations. The fragmentation analysis of both show dominant fragments for *m/*z 146.081 [M-Glc+H_2_O+H]^+^, *m/z* 128.070 [M-Glc+H]^+^, and *m/z* 100.075 [M-Glc+H_2_O-CO_2_+H]^+^ (Figure 6, F and G). The additional low abundant analytical fragment of *m/z* 262.127 represents an NHP-*O*Glc fragment [M-CO_2_+H]^+^ which has lost the carboxy group, however, it has the glucose moiety still attached via the ether-bond. This indicates that UGT76B1 catalyzes the *O*-glycosylation mechanism in vitro and in vivo. Together, our in vitro analysis shows that the purified recombinant UGT76B1 was active toward NHP, SA, and ILA. In addition, we show that UGT76B1 *O-*glycosylates NHP by producing NHP-*O*Glc.

We further analyzed active site residues in enzymes capable of glycosylating SA (UGT74F1 and UGT74F2) and compared them with the UGT76B1 protein sequence (Supplemental Figure S5a). In addition, we made an in silico structural prediction of UGT76B1 using the deposited structure of UGT74F2 (PDB accession 5V2J) (George Thompson et al., 2017) and modeled NHP in the electron density of the co-crystalized SA-analog 2-bromobenzoic acid (Supplemental Figure S5, b and c). Some residues such as histidine at position 20 (His20) and aspartic acid at position 109 (Asp109) that have been shown to be important for the formation of SAG and SGE are conserved in all three UGTs (Supplemental Figure S5a) (George Thompson et al., 2017). However, two threonine residues involved in the glycosylation of SA in UGT74F2 are substituted by leucine at position 17 and glycine at position 363 (Supplemental Figure S5, a and c). Nevertheless, we identified a threonine at position 131 in a predicted loop region, which might compensate for the lack of Thr17 and Thr363 in the catalytic reaction (Supplemental Figure S5, a and c). These findings support our experimental data that the minimum subset of amino acids for fulfilling the glycosylation reactions on SA and NHP are present in UGT76B1’s putative active site.

### Deuterated NHP is translocated to distal tissue

**Figure 7:**
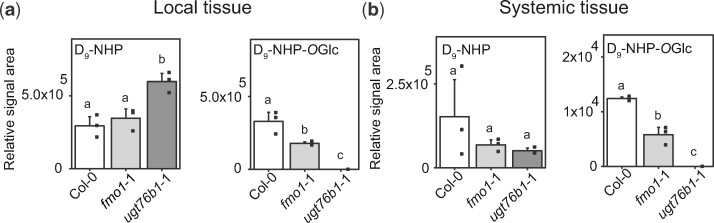
Infiltrated D_9_-NHP moves systemically and is converted to D_9_-NHP-*O*Glc in wild-type and *fmo1*-1 but not in *ugt76b1*-1 plants. Relative signal areas of D_9_-NHP and its glucoside D_9_-NHP-*O*Glc was analyzed 24 h after infiltration of D_9_-NHP to local tissue. Local (A) and systemic (B) leaves were harvested and analyzed by UHPLC-HRMS. Error bars represent standard deviation. Letters indicate statistical differences (*P* < 0.05, one-way ANOVA; *n* = 3). Replicates represent individual pools of 4–6 leaves out of six equally grown and treated plants. The results were confirmed by a second independent experiment.

NHP is the biological active metabolite of Pip in plant defense, especially in SAR (Chen et al., 2018; Hartmann et al., 2018). Nevertheless, it is still an open question whether NHP or NHP-*O*Glc might act as a mobile signal in SAR (Chen et al., 2018; Holmes et al., 2019). To address this question, we infiltrated uniformly deuterated NHP (D_9_-NHP) into leaves of Col-0, *fmo1*-1, and *ugt76b1*-1 plants. Twenty-four hours post-infiltration, local as well as systemic leaves were harvested. First, the formation of D_9_-NHP-*O*Glc from the infiltrated D_9_-NHP in the local leaves of Col-0, *fmo1*-1, and *ugt76b1*-1 plants was monitored by qualitative UHPLC-HRMS. As expected, the applied D_9_-NHP was converted to D_9_-NHP-*O*Glc in the local leaves of wild-type and *fmo1*-1 plants, but no D_9_-NHP-*O*Glc was detected in *ugt76b1*-1 plants (Figure 7). Accordingly, the relative signal area of D_9_-NHP was two times higher in the local leaves of *ugt76b1*-1 plants in comparison to Col-0. Further analysis showed that D_9_-NHP was present in systemic tissue of the three genotypes Col-0, *fmo1*-1, and *ugt76b1*-1, whereas D_9_-NHP-*O*Glc was only detected in Col-0 and *fmo1*-1 plants. This indicates that D_9_-NHP can move to distal tissues without glycosylation.

### *ugt76b1* plants exhibit enhanced resistance in systemic tissue

**Figure 8:**
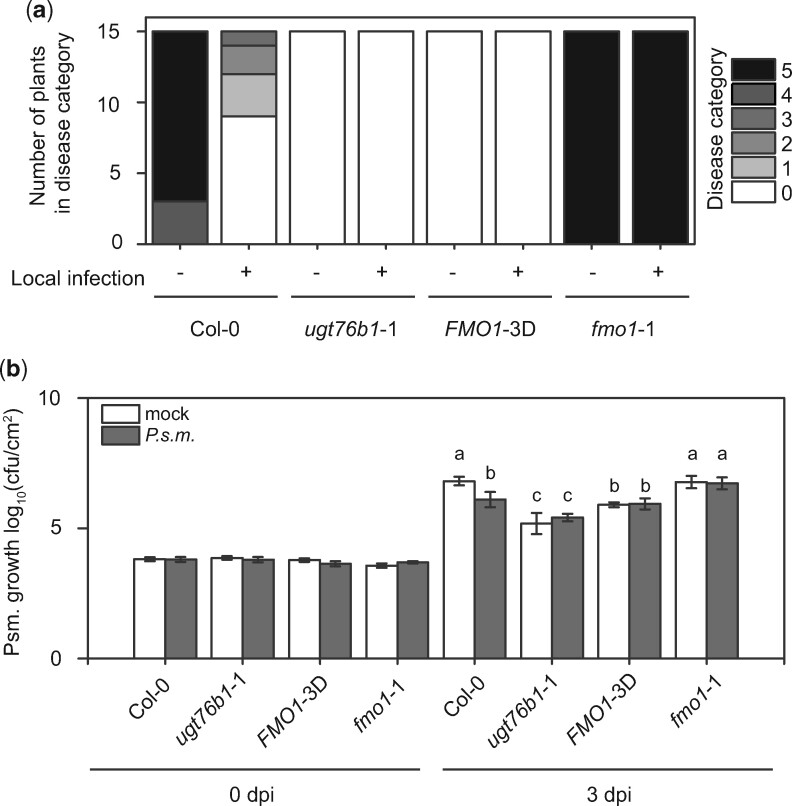
Growth of *H.a.* Noco2 and *P.s.m.* on the distal leaves of wild-type (Col-0), *ugt76b1*-1, *FMO1*-3D, and *fmo1*-1 plants. A, Three-week-old plants were first infiltrated with *P.s.m.* (OD_600_ = 0.001) or 10 mM MgCl_2_ (mock) on two primary leaves and sprayed with *H.a.* Noco 2 spores (5 × 10^4^ spores/mL) 2 days later. Infections on systemic leaves were scored 7 days after inoculation as described previously (Zhang et al., 2010). A total of 15 plants were scored for each treatment. Disease rating scores are as follows: 0, no conidiophores on the plants; 1, one leaf was infected with no >5 conidiophores; 2, one leaf was infected with >5 conidiophores; 3, two leaves were infected but no >5 conidiophores on each infected leaf; 4, two leaves were infected with >5 conidiophores on each infected leaf; 5, >2 leaves were infected with >5 conidiophores. Similar results were obtained in three independent experiments. (B) Four-week-old plants were first infiltrated with *P.s.m.* (OD_600_ = 0.001) or 10 mM MgCl_2_ (mock) on two primary leaves. Two days later, two upper leaves were challenged with *P.s.m.* (OD_600_ = 0.001). Infections on systemic leaves were scored directly after (0 dpi) and 3 days post-inoculation (3 dpi). Error bars represent standard deviation. Letters indicate statistical differences (*P* < 0.05, one-way ANOVA; *n* = 7–8 biological replicates each representing a single infected plant). The results were confirmed by a second independent experiment.

Next, we analyzed whether *ugt76b1*-1 can still establish SAR without the accumulation of NHP-*O*Glc by conducting an *H.a.* Noco 2 growth assay on plants pre-treated with *P.s.m.* (Figure 8). Establishment of SAR strongly reduces the disease rate of distal leaves (indicated as disease categories from 0 to 5) during a second infection with *H.a.* Noco 2, as shown for Col-0 plants (Figure 8A). Plants mock treated on the primary leaf showed high infection rates, indicated by disease categories of four and five on the systemic leaves after plant *H.a.* Noco 2 infection. For *ugt76b1*-1 plants, infection on the systemic leaves was reduced to minimum (disease category 0) regardless of whether they were pre-induced with *P.s.m.* or not. These disease rates were as low as those known for the *FMO1*-3D mutant. In contrast, *fmo1*-1 plants are not able to establish SAR and therefore show an increased susceptibility to *H.a.* Noco2, as known from the literature (Ding et al., 2016). This finding indicates that the distal parts of *ugt76b1*-1, regardless of a primary infection, exhibit enhanced resistance toward *H.a.* Noco 2. This is consistent with results from our local *H.a.* Noco 2 infection assays for the *ugt76b1* lines (Figure 4A). In an independent approach, we analyzed the resistance of *ugt76b1*-1 to a secondary infection by *P.s.m.*. As expected, Col-0 established SAR after primary infection, *fmo1*-1 plants were not able to establish SAR, and *FMO1*-3D showed a constitutive SAR phenotype (Figure 8B). Nevertheless, *ugt76b1*-1 exhibited reduced bacterial growth in distal leaves of both mock and *P.s.m.*-treated samples. Together, these data suggest that *ugt76b1*-1 displays constitutive resistance toward pathogens.

## Discussion

The identification of FMO1 as an NHP biosynthetic enzyme was a major breakthrough toward the understanding of Pip-mediated plant immunity and its involvement in the establishment of SAR (Chen et al., 2018; Hartmann et al., 2018; Holmes et al., 2019). In addition, NHP-*O*Glc was recently described as a metabolite of NHP (Chen et al., 2018). However, the enzyme catalyzing the formation of NHP-*O*Glc was unknown. In this study, we identified UGT76B1 as the enzyme responsible for the glycosylation of NHP in vivo and in vitro—in addition to its previously identified substrates SA and ILA. Besides its glycosyltransferase activity toward NHP in vitro, we show that UGT76B1 is required for the formation of NHP-*O*Glc *in planta* during pathogen infection. The absence of UGT76B1 leads to a significantly increased accumulation of the plant immune regulator NHP, and the complete depletion of NHP-*O*Glc in *ugt76b1* mutant plants. Our data emphasize UGT76B1 as the only enzyme that *O*-glycosylates NHP *in planta*.

*ugt76b1* mutants have been shown to exhibit enhanced disease resistance against biotrophic pathogens, which was suggested to be caused by increased accumulation of SA (Noutoshi et al., 2012). The substrate ILA was recently suggested to activate the immune response via SA by inactivating UGT76B1 (Bauer et al., 2020). In *ugt76b1* mutants, however, NHP accumulates to considerably higher level than in the wild-type during pathogen infection, suggesting that the elevated NHP level, instead, may play a major role in enhancing disease resistance in the mutant plants. This is supported by the complete suppression of the autoimmune phenotype of *ugt76b1* by loss of function of FMO1. The accumulation of NHP leads to dwarfism as reported for the *FMO1*-3D overexpression line. Furthermore, increased NHP levels lead to enhanced resistance of this mutant (Koch et al., 2006). In contrast, the plant size increases as the amount of NHP decreases and its susceptibility toward biotrophic pathogens increases (Figures 4 and 8) (Hartmann et al., 2018). The induction of *UGT76B1* by *P.s.m.* infection therefore suggests that this gene plays a major role in regulating NHP homeostasis, which seems to be critical to balance growth and defense in plants.

Although the NHP level is higher in *ugt76b1* mutants, the increased accumulation of SA is most likely due to the reduced conversion of SA to SAG rather than the effect of NHP on the transcript levels of SA biosynthesis genes (Figure 5). In addition, the *FMO1*-3D mutant does not accumulate free SA to higher levels than the wild-type and a lack of NHP does not affect the accumulation of SA in *fmo1*-1 plants (Koch et al., 2006; Bartsch et al., 2010). The increase of SA and NHP levels in *ugt76b1* mutants suggests that reduced turnover could be a critical mechanism for increasing the accumulation of SA as well as NHP (Supplemental Figure S6).

As there are three UGTs described to glycosylate SA, reduced accumulation of SAG could also hint at a deregulation mechanism in *ugt76b1*-1 plants toward the previously described SA UGTs, especially SAG-forming enzyme UGT74F1 (Dean and Delaney, 2008; George Thompson et al., 2017). The increased basal SGE level in *ugt76b1*-1 has already been addressed and connected to high basal *PR1* expression (von Saint Paul et al., 2011). However, after infiltration with *P.s.m*., transcript levels of *PR1* are similar in Col-0 and *ugt76b1*-1 (Supplemental Figure S3). Furthermore, the transcript levels of *UGT74F2* encoding the SGE forming enzyme were similar in the wild-type and *ugt76b1*-1 mutant. We conclude that the reported increase of SGE after infection of *ugt76b1*-1 is likely caused by the accumulation in UGT74F2’s substrate SA (Figure 1).

ILA was previously identified as a substrate of UGT76B1 (von Saint Paul et al., 2011); however, it was not identified as a molecular marker of infection with *Pseudomonas* in our nontargeted metabolite fingerprinting approach by UHPLC-HRMS (Supplemental Dataset 1). We observed neither ILA accumulation in *ugt76b1-*1, nor the respective glucoside in wild-type plants after infection. Although there might be a chance that our workflow is not sufficient to detect these compounds in vivo, the intracellular concentration of ILA in the shoot was quantified to be approximately 2.5 ng/g dry weight and 7 ng/g dry weight for Col-0 and *ugt76b1*-1, respectively. Estimating a weight loss of at least 1:10 (*m/m*) between dry and fresh weight, the presented amounts of NHP are a multiple of ILA amounts in the shoot. Considering the determined *K*_M_ value of UGT76B1 for NHP in comparison with the one toward ILA presented earlier (472 ± 97 *µ*M), we consider ILA of minor importance for the observed enhanced resistance phenotype (Maksym et al., 2018). Nevertheless, recent data suggest that ILA fulfills its role in controlling NHP and SA glycosylation reactions and therefore has the ability to fine-tune NHP and SA accumulation and their defense amplification loop (Bauer et al., 2020, 2021). This interplay between SA and NHP was additionally shown by Bauer and colleagues, identifying equally high susceptibility of the SA-deficient mutant NahG *sid2* and NahG *sid2 ugt76b1*. These observations suggest that NHP alone is not sufficient to fulfill a robust defense response. All things considered, it is most likely that the enhanced resistance phenotype of *ugt76b1-*1 is due to increased accumulation of NHP and SA.

The catalytic efficiency of UGT76B1 toward SA has been determined to be 2.5-fold higher than for NHP. The determined catalytic efficiency for SA (*k*_cat_/K_M_ = 0.308 s^−1^ mM^−1^) is twice as high as reported by Noutoshi et al. (2012) (*k*_cat_/*K*_M_ = 0.15 s^−1^ mM^−1^) and ∼7 times less than reported by Maksym et al. (2018) (*k*_cat_/K_M_ = 2.2 s^−1^ mM^−1^). Additionally, the *K*_M_-value for SA determined in this work is two times lower (*K*_M_ = 0.075 ± 0.002 mM) compared to earlier reports (Noutoshi et al., 2012; Maksym et al., 2018). Nevertheless, NHP and SA differ in their absolute amount in infected leaf material (Figure 3, A and B) by several orders of magnitude, suggesting that NHP is the more accessible and, therefore, the preferred substrate of UGT76B1. Although an amino acid sequence comparison of UGT74F1 and UGT74F2 with UGT76B1 revealed only 26.96% and 26.75% sequence identity, respectively, two critical residues for glycosylation (His20 and Asp109) in the putative active site are conserved among these UGTs (Supplemental Figure S5) (George Thompson et al., 2017). Interestingly, we were not able to detect glycosylation of 4-hydroxy benzoic acid (4-OH-BA) by UGT76B1 either at the hydroxyl group or the carboxyl group. This suggests that a hydroxyl group in *ortho* or *meta* configuration adjacent to the carboxyl function is important for optimal binding of the ligand in the active site of UGT76B1.

The data presented in Figure 6, F and G indicate that UGT76B1 forms NHP-*O*Glc via an *O*-glycosylation reaction. The analytical fragment of *m/z* 262.129 is specific for NHP-*O*Glc representing [M-CO_2_+H]^+^. The nature of the glycosylation site of NHP at the *N*-hydroxy group by UGT76B1 was additionally confirmed via TMS-derivatization of NHP and transiently produced NHP-*O*Glc in a recent publication (Holmes et al., 2021). Furthermore, NHP derivatives were methylated and analyzed by collision-induced dissociation. The fragments let the authors conclude that glycosylation by UGT76B1 results in NHP-*O*Glc rather than an NHP-*O*Glc-ester (Holmes et al., 2021).

From our transport experiments with D_9_-NHP, we suggest that native NHP rather than NHP-*O*Glc might be a mobile signal that can translocate from the apoplast to the cytosol and move to distal tissue during the establishment of SAR. This may be supported by an earlier study in which SAG was infiltrated into tobacco (*N. tabacum*) leaves (Hennig et al., 1993). Here, the authors showed that SAG was hydrolyzed in the apoplast to SA and that SA rather than SAG entered the cell. In addition, other studies support our notion that both NHP and SA are mobile between local and systemic tissue in Arabidopsis and tobacco (Yalpani et al., 1991; Chen et al., 2018; Lim et al., 2020). Nevertheless, this possibility remains a matter of debate, as evidence has also been presented that SA is not the mobile signal for SAR (Vernooij et al., 1994a, 1994b). However, the formation of SAG and NHP-*O*Glc probably have a central role in inactivating SA and NHP as biologically active molecules, as the dwarf phenotype of the corresponding mutant suggests (Figure 4c) (Noutoshi et al., 2012).

Together, our data extend the NHP metabolic pathway down to NHP-*O*Glc and illustrate the major importance of UGT76B1 in metabolic regulation and maintaining the balance between growth and defense responses.

## Materials and methods

### Plant materials and growth conditions

Plants used for this work are all in the *Arabidopsis thaliana* Col-0 ecotype background. The *fmo1*-1 and *ugt76b1-*1 (SAIL_1171_A11) T-DNA insertion lines were obtained from Nottingham Arabidopsis stock center (NASC; University of Nottingham) and were described previously (Bartsch et al., 2006; von Saint Paul et al., 2011). *ugt76b1-*3 and *ugt76b1-*4 are independent *ugt76b1-*1 deletion lines generated by CRISPR-Cas9 in the Col-0 background, with original lab code of CRISPR UGT #5 and #17, respectively. Double mutant lines *fmo1-*1 *ugt76b1*-40 and *fmo1-*1 *ugt76b1*-104 were generated by crossing *ugt76b1*-1 with *fmo1*-1. In addition, a CRISPR deletion line of *UGT76B1* was generated in the *fmo1*-1 background and is referred to as *fmo1*-1 *ugt76b1*-5. The overexpression mutant *FMO1*-3D was described previously (Koch et al., 2006). Plants were grown for 4–6 weeks under short-day conditions (8-h light/18-h dark cycle) with 100–120 *µ*mol/m^2^/s of light intensity at 80% relative humidity unless specified. The used bulb type was MASTER LEDtube HF 600 mm HO 8W840 T8 (PHILIPS AG, Amsterdam, Netherlands).

### Construction of plasmids for *UGT76b1* gene editing and generation of deletion mutants

Three deletion lines *ugt76b1-*3, *ugt76b1-*4, and *ugt76b1-*5 *fmo1-*1 (original lab code CRISPR UGT #5, CRISPR UGT #17, and CRISPR UGT in *fmo1* #1, respectively) were generated by the CRISPR/Cas9 system as described (Xing et al., 2014). Two single guide RNAs were designed to target *UGT76B1* genomic DNA to generate a approximately 1,000-bp deletion. The PCR fragment containing the guide RNA sequences was amplified from the pCBC-DT1T2 vector with primers 3G11340-BsFF0 and 3G11340-BsRR0 and subsequently inserted into the pHEE401 vector using the BsaI site. The derived plasmid was transformed into *E. coli* and later *Agrobacterium* by electroporation. Col-0 and *fmo1-*1 plants were transformed with the *Agrobacterium* carrying the plasmid by floral dipping (Clough and Bent, 1998). T_1_ plants were screened for deletion mutants by PCR with primers listed in Supplemental Table S1. Homologous deletion mutants were obtained in the T_2_ generation.

### Elicitation of defense response by UV-C and *P.s.m.*

Plants were treated for 20 min with UV-C radiation in a sterile bench (Telstar Bio-II-A, Azbil Telstar Technologies, Barcelona, Spain). The sterile bench was pretreated with UV-C for 20 min prior to radiating the plants. Untreated control plants and the UV-C-treated plants were harvested 24 h later. Infection of plants was conducted by infiltrating plant leaves with *P.s.m.* ES4326 at OD_600_ = 0.05 in 10 mM MgCl_2_, if not stated otherwise, to induce defense. The bacteria were grown in LB medium with Rifampicin (50 *µ*g/µL). In the D_9_-NHP tracking experiment, 82 *µ*g/mL of chemically synthesized D9-NHP was added to the infiltration solution.

### Metabolite extraction

Leaves were harvested 24 hpi and frozen in liquid nitrogen. The samples were ground under liquid nitrogen using Retsch 200 MM (Retsch, Haan, Germany). Ground material was weighed and extracted after a modified methyl-*tert*-butyl ether (MTBE) extraction (Feussner and Feussner, 2019). When metabolite quantification was desired, deuterium-labeled D_9_-NHP and D_6_-SA and isotopically labeled ^13^C-SAG were added prior to extraction. The labeled compound served as a reference throughout the quantitative analysis.

### UPLC-nanoESI-QTRAP-MS-based metabolite quantification

Absolute quantification of NHP, NHP-*O*Glc, SA, and SAG was performed according to a method previously described with the following modifications (Herrfurth and Feussner, 2020). About 100 mg of flash-frozen leaf tissue was ground and subjected to MTBE extraction, including the addition of 50 ng D_9_-NHP (kindly provided by Prof. Ulf Diederichsen, Goettingen, Germany), 10 ng D_4_-SA (C/D/N Isotopes Inc., Pointe-Claire, Canada), and 50 ng ^13^C_6_-SAG (kindly provided by Prof. Petr Karlovsky, Goettingen, Germany). For triple quadrupole linear ion trap (QTRAP)-MS detection, multiple reaction monitoring (MRM) transitions were analyzed as shown in Supplemental Table S2. For quantification, the signal area of each respective authentic standard was compared to the signal area of the analyte of the biological sample. Signals were analyzed in this study when the signal to noise ratio was >20. Furthermore, the determination underlies the previously conducted calibration and technical optimization for each compound. Calibration was carried out using nine data points of varying concentrations for SA, SAG, and NHP-*O*Glc (Supplemental Figure S7, a, b and d). In the case of NHP, calibration was carried out using six data points, due to detection limitations in the low concentration range (Supplemental Figure S7c). Signals were used for calibration purposes when the minimal signal to noise ratio was >8. We determined the limit of detection as for SA (0.007 nmol), SAG (0.003 nmol), NHP (0.034 nmol), and NHP-*O*Glc (0.003 nmol). The limit of quantification was determined at a signal to noise ratio of eight in plant extract matrices for SA (0.013 nmol/100 mg), SAG (0.7 nmol/100 mg), NHP (1.03 nmol/100 mg), and NHP-*O*Glc (0.05 nmol/100 mg). D_9_-NHP was synthesized as described previously (Hartmann et al., 2018). Synthesized NHP was characterized via tandem MS (MS/MS) fragmentation (Rekhter et al., 2019a). The fragmentation behavior underlying the MRM transitions of NHP-*O*Glc were analyzed after thin-layer chromatographic purification of enzymatically produced NHP-*O*Glc using UGT76B1. As stationary phase, a TLC silica gel 60 (Merck KGaA, Darmstadt, Germany) was used in combination with butanol:water:acetic acid (4:1:1, *v/v/v*) as the solvent system (Song, 2006). Purified NHP-*O*Glc was extracted from the silica gel with MTBE corresponding to the extraction procedure as described (Herrfurth and Feussner, 2020). Successful purification of enzymatically produced NHP-*O*Glc was checked via nontargeted UHPLC-HRMS. The purified NHP-*O*Glc was quantified by direct infusion-MS with respect to SAG (kindly provided by Prof. Petr Karlovsky, Goettingen, Germany).

### UHPLC-HRMS-based metabolite fingerprint analysis

Metabolites were extracted from 100 mg leaf material by two-phase extraction with MTBE, methanol, and water according to Feussner and Feussner (2019). Metabolite fingerprint analysis of the metabolites of the polar extraction phase was performed with the UHPLC1290 Infinity (Agilent Technologies, Santa Clara, CA, USA) coupled to an HRMS instrument (6540 UHD Accurate-Mass Q-TOF, Agilent Technologies, Santa Clara, CA, USA) with Agilent Dual Jet Stream Technology as electrospray ionization (ESI) source (Agilent Technologies, Santa Clara, CA, USA). For chromatographic separation, an ACQUITY HSS T3 column (2.1 × 100 mm, 1.8 μm particle size, Waters Corporation, Milford, MA, USA) was used with a flow rate of 500 µL/min at 40°C. The solvent systems A (water, 0.1% (*v/v*) formic acid) and B (acetonitrile, 0.1% (*v/v*) formic acid) were used for the following gradient elution: 0–3 min: 1–20% B; 3–8 min: 20–97% B; 8–12 min: 100% B; 12–15 min: 1% B. The quadrupole time of flight (QTOF) MS instrument was used in a range from *m/z* 50 to *m/z* 1700 with a detection frequency of 4 GHz, capillary voltage of 3000 V, and nozzle and fragmentor voltage of 200 and 100 V, respectively. The sheath gas was set to 300°C, and gas to 250°C. The gas ﬂow of drying gas was set to 8 L/min and sheath gas to 8 L/min, respectively. Data were acquired with Mass Hunter Acquisition B.03.01 (Agilent Technologies, Santa Clara, CA, USA) in positive as well as ESI mode. For data deconvolution, the software Profinder B.08.02 (Agilent Technologies, Santa Clara, CA, USA) was used. For further data processing, statistics, data mining, and visualization, the tools of the MarVis-Suite (Kaever et al. 2015, http://marvis.gobics.de/) were applied. Overall, 448 metabolite features (307 features from positive and 141 features from negative ESI mode) with an FDR < 0.005 were selected and clustered by means of one-dimensional self-organizing maps. The accurate mass information of the metabolite features was used for metabolite annotation (Kyoto encyclopedia of genes and genomes [KEGG], http://www.kegg.jp and BioCyc, http://biocyc.org, in-house database). The chemical structure of the indicated metabolites was confirmed by LC-HRMS/MS analyses (NHP: [M + H]^+^ 146.080, 128.070, 110.06, 100.076, 82.065, 70.065, and 55.055 (Rekhter et al., 2019b); NHP-*O*Glc: [M + H]^+^ 308.132, 146.081, 128.0705, 110.06, 100.076, 82.062, 70.065, and 55.055 (Rekhter et al., 2019b); SA: [M−H]^−^ 137.025 and 93.035 (METLIN, https://metlin.scripps.edu/), MID3263); SAG: [M−H]^−^ 299.0719, 137.024, and 93.035; Pip: [M + H]^+^ 130.086, 84.081, 70.065, and 56.050 (Ding et al., 2016); 2HNG: [M−H]^−^ 216.051, 172.062, 128.072, and 86.025 (Rekhter et al., 2019b) and SGE: [M−H]^−^ 299.078, 137.024, and 93.035). The results were confirmed by two independent experiments with three biological replicates each.

### RNA extraction, reverse transcription, and qPCR

Plants for gene expression assays were grown on soil under long-day (16-h light) conditions. Three leaves of 4-week-old plants (∼50 mg) were collected for RNA extraction using an EZ-10 Spin Column Plant RNA Miniprep Kit (Bio Basic Inc., Toronto, Canada). RNAs were then reverse transcribed into cDNAs by OneScript Reverse Transcriptase (Applied Biological Materials Inc., Richmond, Canada). qPCR was performed with cDNAs using SYBR Premix Ex Taq™ II (Takara, Shiga, Japan). For pathogen-induced gene expression assays, plants were grown under short-day (12 h light) conditions. Three leaves of 4–6-week-old plants were infiltrated with *P.s.m.* (OD_600_ = 0.001). Leaves were harvested at 24 hpi and analyzed via the process described above. Primers for qPCR are listed in Supplemental Table S1.

### Heterologous protein expression and purification

His-tagged UGT76B1 was purified via a combination of methods described recently (Maksym et al., 2018; Haroth et al., 2019). UGT76B1 (*AT3G11340*, GenBank Accession Number Q9C768.1) was amplified from total cDNA derived from infected leaf tissue and cloned into the pET28a vector (Merck, Darmstadt, Germany) using the BamHI and SalI restriction sites. The plasmid containing the *UGT76B1* gene was transformed into BL21 Star (DE3) cells (Thermo Fisher Scientific, Waltham, MA, USA) by heat shock. Cell cultures were grown in auto-induction medium (Studier, 2005) at 16°C for 4 days. Cell pellets of a 1 L culture were resuspended in lysis buffer (50 mM Tris/HCl pH = 7.8, lysozyme, DNAseI, and 0.1 mM PMSF). After homogenization, cells were disrupted by ultrasonication. Cleared lysate was obtained by centrifugation at 25,000 ×g for 45 min at 4°C. The recombinant protein was purified from the cleared lysate using a combination of metal affinity chromatography using nickel-affinity (GE Healthcare, Chicago, IL, USA) and size exclusion chromatography using 16/600 Superdex 75 prep grade columns (GE Healthcare, Chicago, IL, USA).

### Liquid chromatography (LC)-MS-based activity assay and in vitro kinetics

UGT76B1 recombinant protein was incubated with substrates NHP, SA, and ILA for 30 min at 30°C. The reaction was stopped by the addition of 20% acetonitrile. Samples were analyzed using a 1290 Infinity UHPLC system coupled to a 6540 UHD Accurate-Mass Q-TOF (Agilent Technologies, Santa Clara, CA, USA) as previously described (Feussner and Feussner, 2019). Kinetic parameters of UGT76B1’s substrates NHP, SA, and ILA were analyzed as described under UPLC-nanoESI-QTRAP-MS-based metabolite quantification. The reaction mixture contained 3.5-*µ*g UGT76B1, 2 mM UDP-Glc (Merck, Darmstadt, Germany), and 0–2.5 mM substrate. Before incubation with UGT76B1, the initial amount of substrate was determined for analysis of substrate reduction. The reaction was incubated for 15 min at 30°C and stopped by the addition of methanol. The difference in signal intensity of substrate was plotted for each substrate and concentration. The *K*_M_ was determined via Hill regression analysis using OriginPro version 8.5 (OriginLab Corporation, Northampton, MA, USA).

### Pathogen infection assay and SAR assay

Basal resistance against *H.a.* Noco 2 was tested by spray-inoculating 2-week-old seedlings with spore solution (50,000 spores/mL). Inoculated seedlings were covered with a transparent lid and grown in a plant chamber with a relative humidity of ∼80%. Infection was scored at 7 dpi by counting conidia spores with a hemocytometer.

Induction of SAR against *H.a.* Noco 2 was performed by infiltrating two full-grown leaves of 3-week-old plants with *P.s.m.* ES4326 (OD_600_ = 0.001) or 10 mM MgCl_2_ (mock). Two days later, plants were sprayed with *H.a.* Noco 2 spore solution (50,000 spores/mL). Infection on distal leaves was scored at 7 dpi as described previously (Ding et al., 2016).

Induction of SAR against *Pseudomonas* was tested by infiltrating *P.s.m.* ES4326 (OD_600_ = 0.001) or 10 mM MgCl_2_ (mock) on two leaves of 4-week-old plants grown under short-day conditions. Two days later, two distal leaves were challenged with *P.s.m.* ES4326 (OD_600_ = 0.001). Infection was scored at both 0 and 3 dpi by measuring the bacterial titer in the distal leaves.

### Structural prediction and ligand docking

The crystal structure of UGT74F2 (George Thompson et al., 2017), co-crystalized with SA-analog 2-bromobenzoic acid, UDP, 3-*O*-β-d-glucopyranosyl-β-d-glucopyranose, and β-d-glucose (PDB ID 5V2J) was used for structural prediction of UGT76B1. The structural prediction of UGT76B1 was done using PHYR2Protein (Kelley et al., 2015). NHP was fit into the electron density of SA-analog 2-bromobenzoic acid using Coot (Emsley and Cowtan, 2004). Figures were created and distances were measured using PyMol (Schrödinger LLC, New York, NY, USA).

### Statistical analysis

Statistical analyses were performed using Origin Pro version 8.5 and Origin 2020 (OriginLab Corporation, Northampton, MA, USA). Results of the one-way-analysis of variance (ANOVA) calculations is provided in the Supplemental Dataset 2.

### Accession numbers

Further deposited information can be found in The Arabidopsis Information Resource database under the accession numbers: AT3G11340 (*UGT76B1*) and AT1G19250 (*FMO1*). Mass spectrometric data of the underlying study have been deposited to Metabolights public repository (Haug et al., 2020) under the study MTBLS2334.

## Supplemental data

The following materials are available in the online version of this article.

**Supplemental Figure S1**. CRISPR deletion mutants of *UGT76B1* are unable to synthesized NHP-*O*Glc after UV-treatment.

**Supplemental Figure S2**. *fmo1*-1 *ugt76b1*-1 double loss-of-function mutant plants synthesize neither NHP nor NHP-*O*Glc after UV-treatment.

**Supplemental Figure S3**. Transcripts levels of *PR1* and *PR2* after infection with *P.s.m*. in *ugt76b1* and wild-type.

**Supplemental Figure S4**. Purification of UGT76B1 heterologously expressed in *E. coli*.

**Supplemental Figure S5**. Modeling of NHP into the SA-analogs electron density in the predicted in silico UGT76B1 model.

**Supplemental Figure S6**. Transcripts of *UGT76B1* were not present in the mutant.

**Supplemental Figure S7**. Calibration linearity as basis for quantification of SA, SAG, NHP, and NHP-*O*Glc.

**Supplemental Table S1**. List of primers used in this work

**Supplemental Table S2**. MRM parameters for absolute quantification of analytes.

**Supplemental Dataset 1.** Data matrix of 448 high-quality metabolite features (FDR < 0.005) obtained by metabolite fingerprinting (UHPLC-HRMS analysis) of mock and *p*.*s*.*m*. infected Col-0 and ugt76b1 plants.

**Supplemental Dataset 2.** ANOVA results underlying figure assignments.

## Supplementary Material

koaa045_Supplementary_Data

## Notes

Y.Z. and I.F. designed and supervised the study. Experimental research was conducted by L.M., D.R., W.H., K.F., and H.T., and C.H. L.M., D.R., W.H., K.F., C.H., Y.Z., and I.F. analyzed the data and wrote the manuscript.

The authors responsible for distribution of materials integral to the findings presented in this article in accordance with the policy described in the Instructions for Authors (https://academic.oup.com/plcell/pages/General-Instructions) are: Yuelin Zhang (yuelin.zhang@ubc.ca) and Ivo Feussner (ifeussn@uni-goettingen.de).
