# Supplementary material for: The glycosyltransferase UGT76B1 modulates N-hydroxy-pipecolic acid homeostasis and plant immunity
Source: Plant Cell. 2021 Jan 11;33(3):735–49. doi: 10.1093/plcell/koaa045 (PMC8136917; doi:10.1093/plcell/koaa045)
Supplement: koaa045_Supplementary_Data [file koaa045_supplementary_data.zip › tpc.00514.2020-s01.pdf]

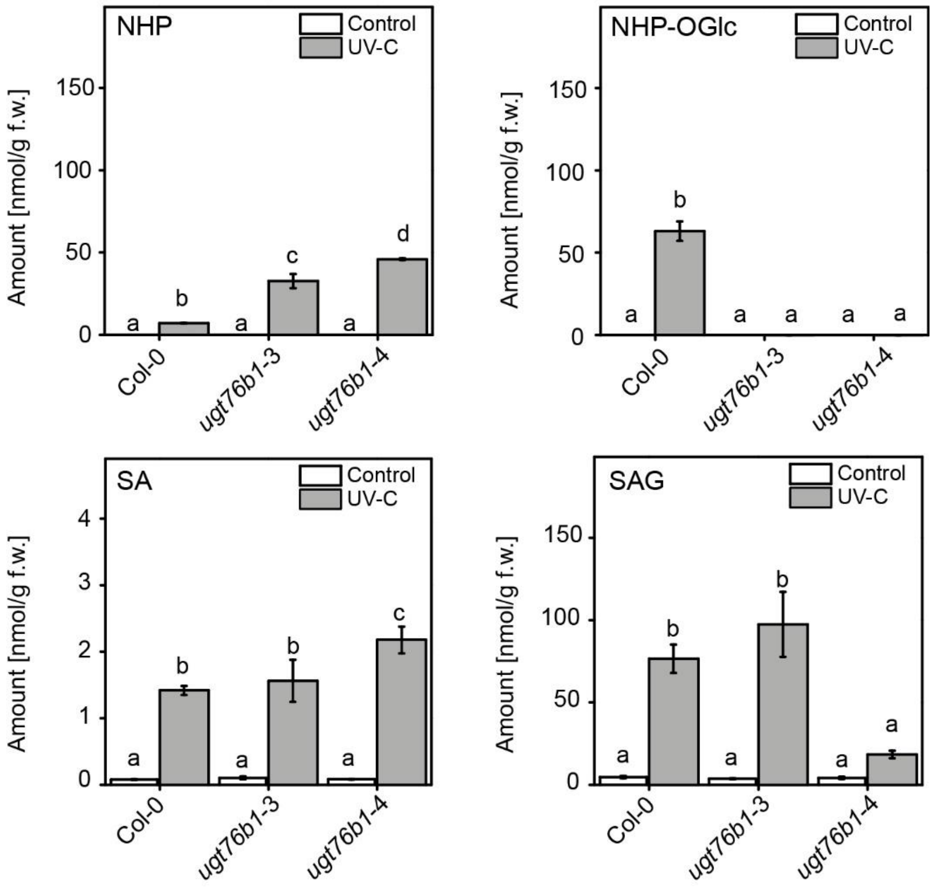

Supplemental Figure 1. **CRISPR deletion mutants of *UGT76B1* are unable to synthesize NHP-*O*Glc after UV-treatment.** Supports Figure 3. Absolute amounts of NHP, NHP-*O*Glc, SA and SAG were determined in wild type, *ugt76b1*-3 and *ugt76b1*-4 after UV-C treatment. Plants grown under long day conditions (16 hours light period), were treated for 20 min with UV-C or left untreated as control. Twenty-four hours post treatment, leaf material was harvested and analyzed using quantitative UPLC-nanoESI-QTRAP-MS. Error bars represent standard deviation. Letters indicate statistical differences ($p < 0.05$, one-way ANOVA; n=3). Replicates represent a pool of 4–6 leaves of 6 plants per condition.

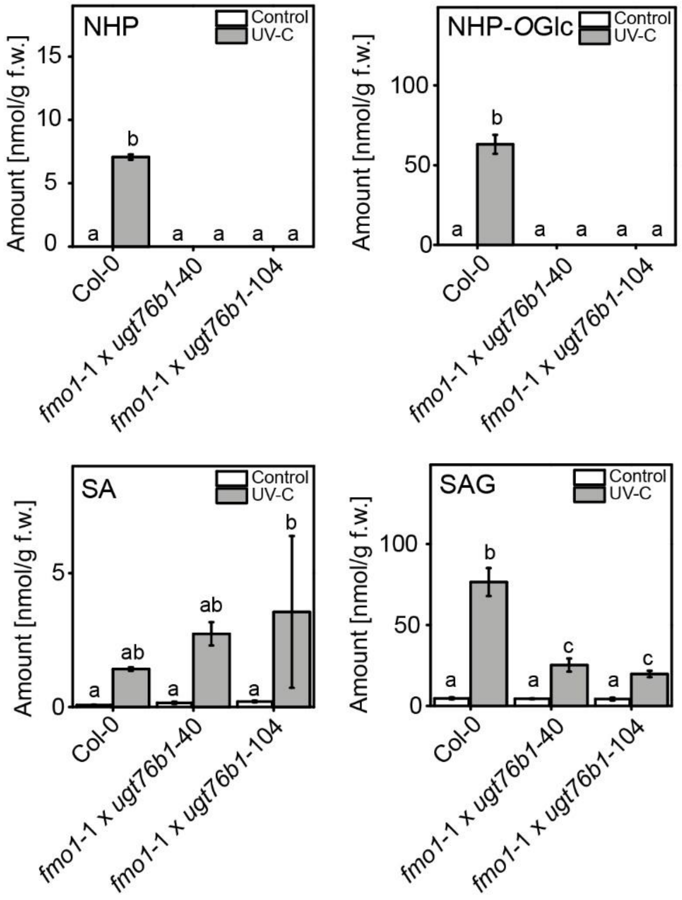

Supplemental Figure 2. ***fmo1*-1 *ugt76b1*-1 double loss-of-function mutant plants synthesize neither NHP nor NHP-*O*Glc after UV-treatment.** Supports Figure 4. Absolute amounts of NHP, NHP-*O*Glc, SA and SAG were determined in wild type and two independent *fmo1*-1 *ugt76b1* lines after UV-C treatment. Plants grown under long day conditions (16 hour light period), were treated for 20 min with UV-C or left untreated as control. Twenty-four hours post treatment, leaf material was harvested and analyzed using quantitative UPLC-nanoESI-QTRAP-MS. Error bars represent standard deviation. Letters indicate statistical differences ($p < 0.05$, one-way ANOVA; n=3). Replicates represent a pool of 4–6 leaves of 6 plants per condition.

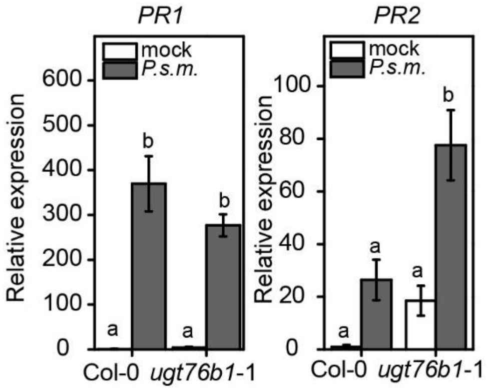

Supplemental Figure 3. **Transcript levels of *PR1* and *PR2* after infection with *P.s.m*. in *ugt76b1* and wild type.** Supports Figure 5. Relative amount of transcripts of *PR1* and *PR1* was analyzed in wild type and *ugt76b1*-1 plants after infection with *P.s.m.* ES4326. Three leaves of 4-6 week-old plants were treated with *P.s.m.* ES4326 (OD_{600}=0.001). Leaves were harvested 24 hours post infiltration and analyzed for the level of transcripts via qPCR. Error bars represent standard deviation. Letters indicate statistical differences ($p <$ 0.05, one-way ANOVA; n=3). Replicates represent a pool of 4–6 leaves of 6 plants per condition.

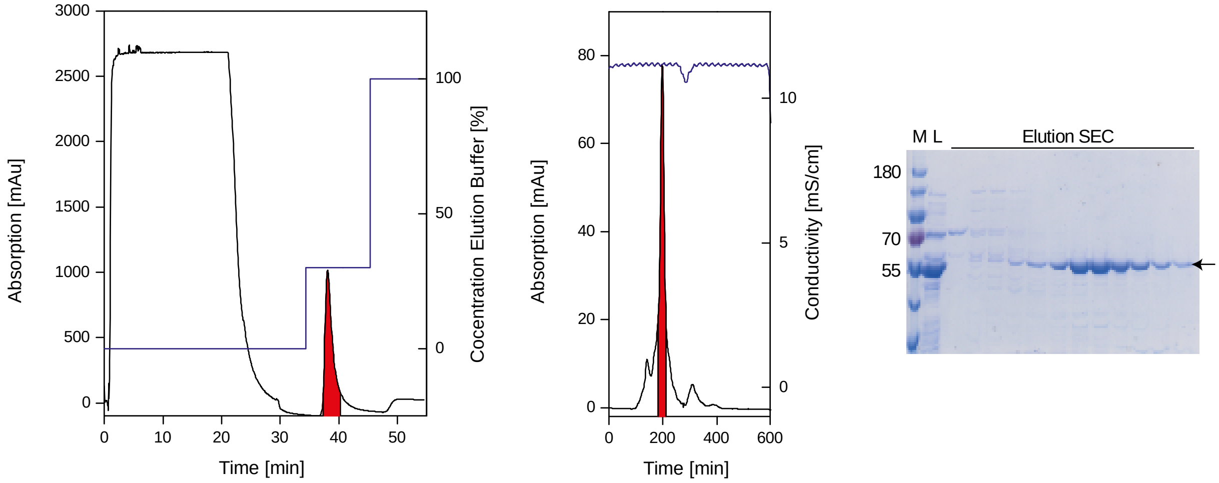

Supplemental Figure 4. **Purification of UGT76B1 heterologously expressed in *E. coli*.** Supports Figure 6. UGT76B1 fused with an N-terminal His-tag was heterologously expressed in *E. coli* BL21 Star (DE3) and purified via a combination of immobilized metal affinity chromatography (IMAC) and size exclusion chromatography (SEC). Chromatograms illustrate the absorption at 280 nm in milli absorption units (mAU) during protein elution. Secondary y-axes indicate the concentration of elution buffer in % for IMAC or the conductivity in mS/cm for SEC. Red areas represent corresponding signals to UGT76B1. The sodium dodecyl sulfate polyacrylamide gel electrophoresis (SDS-PAGE) gel shows the corresponding protein marker M, the load L (eluate IMAC) and the elution after SEC. The arrow indicates UGT76B1. The depicted purification is representative of at least three independent purifications.

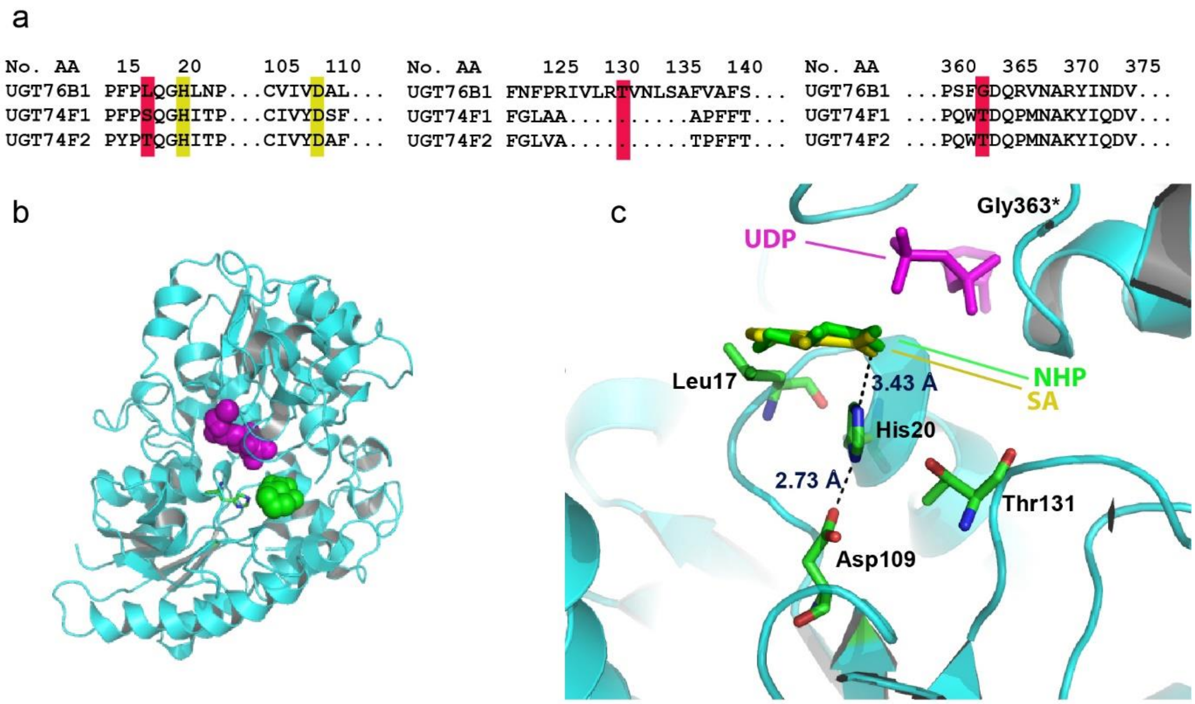

Supplemental Figure 5. **Modeling of NHP into the SA-analogues' electron density in the predicted *in silico* UGT76B1 model.** (supports Figure 6) **(a)** Protein sequence alignment comparing UGT76B1, UGT74F1 and UGT74F2 with the putative active site residues. Sequence identities are shown in yellow and miss matches in red. **(b)** Predicted model of UGT76B1 complexed with UDP and NHP using the deposited PDB structure 5V2J of UGT74F2 complexed with UDP and SA. UDP is shown as magenta balls and the modeled NHP is shown as green balls. His20 is shown as sticks. **(c)** Amino acids histidine (His^{20}), aspartate (Asp^{109}) and putatively threonine (Thr^{131}), which may form the proposed catalytic triad by George Thompson et al., 2017, are predicted to the active center and in close proximity to the substrate and each other in the UGT76B1 model prediction. The structural prediction of UGT76B1 was done by PHYR2Protein (Kelley et al., 2015). NHP was fit into the electron density of SA-analogue 2-bromobenzoic acid using Coot (Emsley and Cowtan, 2004). Figures were created using PyMol (Schrödinger LLC, USA).

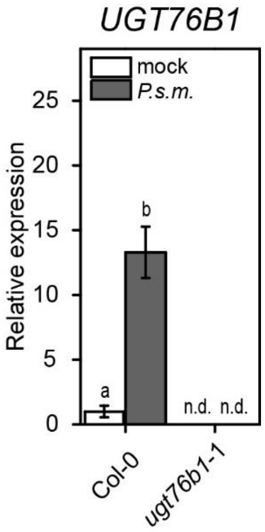

Supplemental Figure 6. **Transcripts of *UGT76B1* were not present in the mutant.** Supports Figures 1, 2, 3, 4, 5 and 7. Relative amounts of *UGT76B1* transcripts were analyzed in wild type and *ugt76b1*-1 plants after infection with *P.s.m.* ES4326. Three leaves of 4–6 week-old plants were treated with *P.s.m.* ES4326 (OD_{600}=0.001). Leaves were harvested 24 hours post infiltration and analyzed for the level of transcripts via qPCR. Error bars represent standard deviation. Letters indicate statistical differences ($p < 0.05$, one-way ANOVA; n=3). Replicates represent a pool of 4-6 leaves of 6 plants per condition.

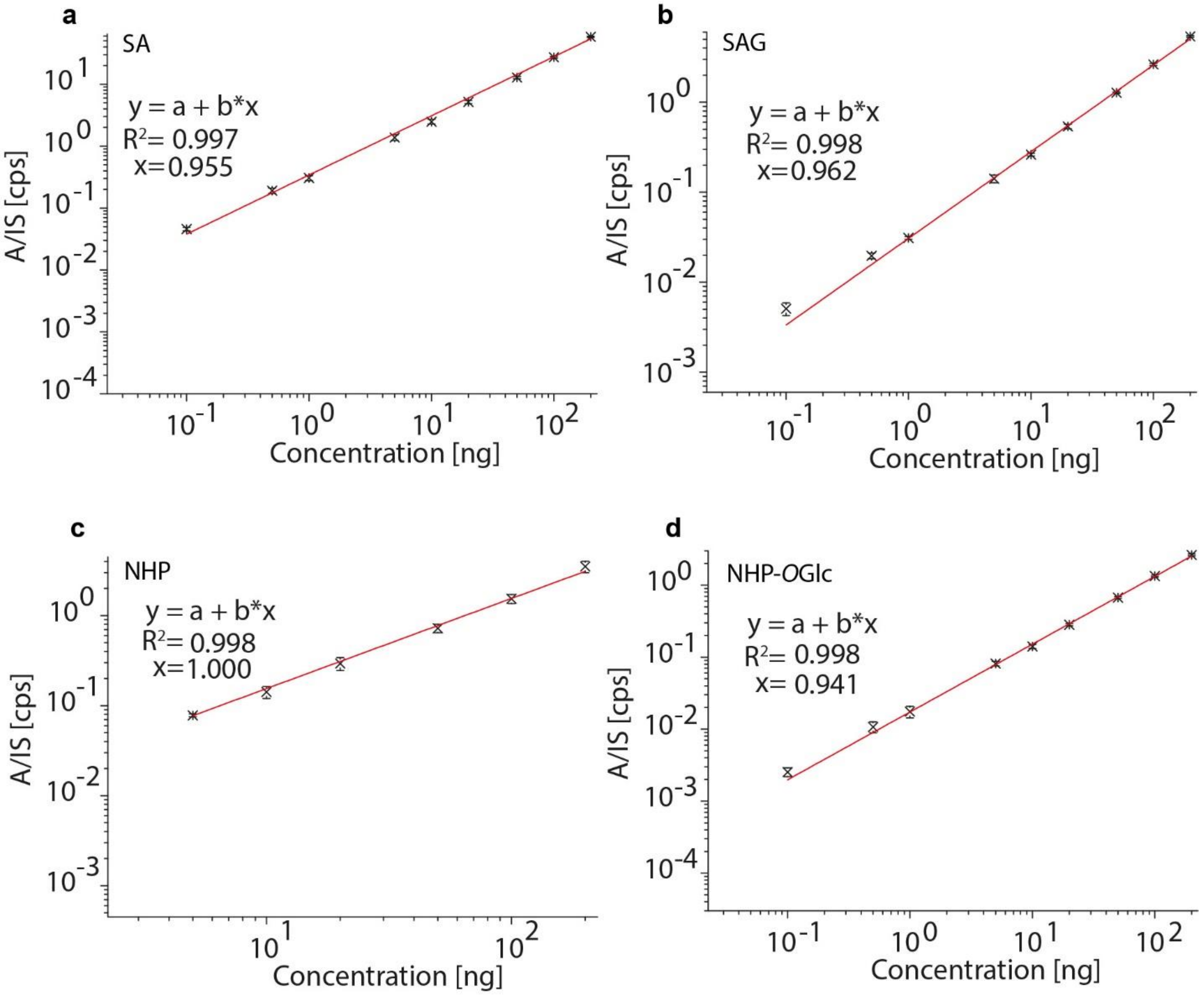

Supplemental Figure 7. **Calibration linearity as basis for quantification of SA, SAG, NHP, NHP-*O*Glc.** Supports Figure 3. Each substance was analyzed at different concentrations to determine the calibration linearity of the UPLC-nanoESI-QTRAP-MS-based method. Six to nine data points were collected for each compound. The data represent the counts per seconds (cps) of the analyte divided by the cps of the internal standard over the concentration dependency in ng of the substance. The data are shown in a $\log_{10}$ scale on both axes. *n*=5 technical replicates of each data point.

Supplemental Table 1. **List of primers used in this work.** Information is divided by primer application for quantitative PCR analysis, genotyping, and cloning.

Real time PCR-primers

Gene ID	Forward primer	Reverse primer
AT2G37620	ACT1-F: cgatgaagctcaatccaaacga	ACT1-R: cagagtcgagcacaataccg
AT2G14610	PR1-RT-F2: AGGCAACTGCAGACTCATAC	PR1-RT-R2: TTGTTACACCTCACTTTGGC
AT4G39030	EDS5-F101-RT: GCCAAACAGGACAAGAAAGAAG	EDS5-R102-RT: GCCGAAACAATCTGTGAAGC
AT5G13320	PBS3-F101-RT: CTAAGTTCTGGAACTTCTGG	PBS3-R102-RT: CATGACTGAAGCAAAGATGG
AT2G13810	ALD1-F101-RT: TTCCCAAGGCTAGTTTGGAC	ALD1-R102-RT: GCCTAAGAGTAGCTGAAGACG
AT1G19250	FMO1-F101-RT: GGAGATATTCAGTGGCATGC	FMO1-R102-RT: TTTGGTTAGGCCTATCATGG
AT1G73805	SARD1-RT-NF: TCAAGGCGTTGTGGTTTGTG	SARD1-RT-NR: CGTCAACGACGGATAGTTTC
AT3G11340	11340-RT-F: GGATTGTTCTCCGAACCGTTA	11340-RT-R: GTGAGTCTGCCTTAGTCTCTTG

Genotyping primers

Lines	Forward primer	Reverse primer
CRISPR ugt76b1 lines	11340-heter-F: GATCGAATCAGCATAATG	11340-heter-R: GTGTCTGATTATGGGAATGC
CRISPR ugt76b1 lines	11340-homo-F: GAATGAAGGATCTTCCATGG	11340-heter-R: GTGTCTGATTATGGGAATGC
SAIL_1171_A11	SAIL1171A11-tdna-F: TCAGGAATCATATTCAACGCC	SAIL1171A11-tdna-R: GCTGAAGACTAAGCGTCATGC

Cloning primers

Purpose	Primer	Sequence
CRISPR-deletion (*UGT76B1*)	3G11340-BsFF0	ATATATGGTCTCGATTG TCTTCCCTTTCCCTTTA CAGTTTTAGAGCTAGAA ATAGC
CRISPR-deletion (*UGT76B1*)	3G11340-BsRR0	ATTATTGGTCTCGAAAC CTTCCGAGCTCGTCATT AGCAATCTCTTAGTCGA CTCTAC
Heterologous expression (*UGT76B1*)	UGT76B1 BamHI for	acgGGATCCATGGAGAC TAGAGAAACA

Heterologous expression (*UGT76B1*)	UGT76B1 SalI reverse	acgGTCGACTTAGAAAG ACAATATATAAGCA

Supplemental Table 2. **Multiple reaction monitoring parameters for absolute quantification of analytes.** For the presented quantitative plant hormone data we established a multiple reaction monitoring analysis of seven additional analytes to the ones published before (Herrfurth and Feussner, 2020). Q1 (precursor ion), Q3 (product ion) and the retention time (RT) of each analyte are shown, respectively. Furthermore, the declustering potential (DP), entrance potential (EP), collision energy (CE) and the cell exit potential (CXP) of each compound are provided.

Q1 (*m*/*z*)	Q3 (*m*/*z*)	RT (min)	Analyte	DP (eV)	EP (eV)	CE (eV)	CXP (eV)
137	93	2	SA	-25	-6	-20	-10
141	97	3	D_4-SA	-25	-6	-22	-6
144	82	0.7	NHP	-60	-8	-15	-13
153	90	0.7	D_9-NHP	-60	-8	-15	-13
299	137	1	SAG	-30	-4	-18	-2
305	137	1	$^{13}C_6$-SAG	-30	-4	-18	-2
306	89	0.9	NHP-*O*Glc	-65	-4	-18	-13
