# Supplementary material for: The glycosyltransferase UGT76B1 modulates N-hydroxy-pipecolic acid homeostasis and plant immunity
Source: Plant Cell. 2021 Jan 11;33(3):735–49. doi: 10.1093/plcell/koaa045 (PMC8136917; doi:10.1093/plcell/koaa045)
Supplement: koaa045_Supplementary_Data [file koaa045_supplementary_data.zip › tpc.00514.2020-s05.pdf]

The glycosyltransferase UGT76B1 modulates *N*-hydroxy-pipecolic acid homeostasis and plant immunity

Lennart Mohnike, Dmitrij Rekhter, Weijie Huang, Kirstin Feussner, Hainan Tian, Cornelia Herrfurth, Yuelin Zhang and Ivo Feussner

Corresponding author: Ivo Feussner ifeussn@uni-goettingen.de

Review timeline:

**TPC2020-RA-00514**	Submission received:	July 1, 2020
	1st Decision:	August 24, 2020 *revision requested*
**TPC2020-RA-00514R1**	1st Revision received:	September 10, 2020
	2nd Decision:	November 12, 2020 *accept with minor revision*
**TPC2020-RA-00514R2**	2nd Revision received:	November 17, 2020
	3rd Decision:	December 10, 2020 *acceptance pending, sent to sci editor*
	Final acceptance:	December 21, 2020

REPORT: (The report shows the major requests for revision and author responses. Minor comments for revision and miscellaneous correspondence are not included. The original format may not be reflected in this compilation, but the reviewer comments and author responses are not edited, except to correct minor typographical or spelling errors that could be a source of ambiguity.)

TPC2020-RA-00514 1st Editorial decision – *revision requested* **August 24, 2020**

We have received reviews of your manuscript entitled "The glycosyltransferase UGT76B1 is critical for plant immunity as it governs the homeostasis of N-hydroxy-pipecolic acid." Thank you for submitting your best work to The Plant Cell. The editorial board agrees that the work you describe is substantive, falls within the scope of the journal, and may become acceptable for publication, pending revision and potential re-review.

We ask you to pay attention to the following points in preparing your revision:

There are some questions about the biochemical assay and if the data is absolute or relative quantification. If the controls can be provided to keep it as absolute that would be great. Alternatively, it would be fine to take it to relative and adjust any potential flux related claims accordingly.

There are also some other suggestions for improvement.

As a side question, some of the requests are actually covered in the other two articles on the same topic and they are proceeding at a similar pace. Would it be acceptable to share manuscripts and use the other manuscripts to cover requests and likewise the other authors could cite your work to cover some of their requests. Would this be acceptable? --- Reviewer comments:

TPC2020-RA-00514R1 1st Revision received **September 10, 2020**

Reviewer comments and **author responses**:

<u>Reviewer #1:</u>

This topic of this study, the glycosylation of N-hydroxypipecolic acid (NHP) and its role in plant defense responses, is currently a very hot topic, as evidenced by the deposition of three independent manuscripts to the bioRxiv preprint server within a two-week time frame in early July. The conclusion of these three manuscripts is the same: the glucosyltransferase UGT76B1 of Arabidopsis is likely to play an important role in the breakdown of NHP and the

response to pathogen attack. The present manuscript by Mohnike et al. reports on very well designed experiments that provide ample evidence for the function of UGT76B1 in these processes (brief bullet point summary):

- Increased accumulation of NHP in loss-of-function mutant (ugt76b1-1) challenged with Pseudomonas.

- Decreased of accumulation of NHP-glucoside to undetectable levels in ugt76b1-1 challenged with Pseudomonas.

- Demonstration that UGT76B1 acts downstream of the previously identified flavin-dependent monooxygenase FMO1 (involved in NHP biosynthesis).

- Increased turnover of NHP and not higher biosynthesis correlate with increased pathogen resistance in ugt76b1-1.

- NHP appears to be viable substrate of UGT76B1 based on Km value in in vitro assays.

- Feeding of several Arabidopsis genotypes with deuterated NHP indicated increase levels of deuterated NHP in ugt76b1-1 local and systemic leaves; deuterated NHP-glucoside was only detectable in control plants.

- Demonstration that UGT76B1 loss of function enhanced pathogen resistance in systemic leaves.

The manuscript is very well written and the approaches are appropriate to address the relevant questions regarding the function of UGT76B1. There are only a few experimental shortcomings that need to be addressed.

Point 1. Quantitative values are presented for the concentrations of several metabolites based on UHPLC-HRMS assays. However, the validation of the underlying method is not presented. This includes determining the recovery of analytes during the extraction process and the limit of detection/quantitation, evaluating the precision of the method, assessing matrix effects in the ion source, and showing calibration curves with authentic standards. Unfortunately, the lack of proper method validation has led to a lot of confusion in the literature, with quantitative values in some cases differing by orders of magnitude. I am including a link to a very helpful guide on quantitative analyses, which summarizes important considerations that have been agreed upon by various learned societies in the analytical sciences: https://www.rsc.org/images/AMC%20LCMS%20Guide_tcm18-240030.pdf. The authors might be fully aware of what needs to be done, in which case they can safely ignore the guide.

> **RESPONSE: We appreciate the valuable comments and we fully agree with the reviewer. Your suggestions encouraged us to show all data and analyses done for method validation in respect to the absolute quantification of NHP, NHP-OGlc, SA and SAG, shown in Figure 3. Please find a more detailed description of the method in the material and method section. We included information about the limits of detection as well as the limits of quantification in Arabidopsis leaf extract. Furthermore, we added additional information about calibration curves (Figure S7).**

Point 2. The kinetic evaluation of UGT76B1 is incomplete as only Km values are given. The authors should also determine Kcat values and then compare Kcat/Km to determine the catalytic efficiency.

> **RESPONSE: We appreciate this comment. For the determination of Km the enzymatic activity of UGT76B1 had been determined via LC-HRMS-based end-point analysis of NHP-OGlc formation. Unfortunately, we are not able to do absolute quantification for NHP-OGlc on our LC-HRMS device (because of a limitation in the availability of our NHP-OGlc standard). Consequently, we could not calculate kcat values and subsequently kcat/Km to determine the catalytic efficiency of UGT76B1.**

<u>Reviewer #2:</u>

In identifying a UGT that glycosylates (and likely inactivates) NHP, this paper makes an important contribution to the field of plant immunity. I particularly liked the elegant combination of genetics and biochemistry. The paper is well written and the experimental results appear to be sound. I have only a few minor points for consideration.

Point 1. For readability by non-specialists, a general biosynthetic scheme (similar to Fig. 8) could be included as Fig. 1. This would help to orient the reader in the Introduction and early Results section. This would not preclude keeping the current proposed pathway in Figure 8. However, it's not clear what is new in Figure 8 other than the identification of UGT76B1. And so perhaps the current Fig 8 can simply be moved to Fig 1, with slight alterations about what was unknown before this study.

RESPONSE: We appreciate this suggestion. We changed the general biosynthetic scheme (previous Fig. 8) to Fig. 1. We used it now in the introduction of the revised manuscript to introduce the pathway and ensure better readability for non-specialists.

Point 2. I was curious to know the confidence with which NHP-O-Glc was identified by LC-MS. Is accurate mass analysis sufficient in the absence of authentic standard? Can it be excluded that Glc is added to the carboxyl group of NHP?

RESPONSE: Thank you for bringing up this point about the confidence in NHP-OGlc identification. The way we described it was indeed not precise enough. In our revised version we now added the LC-HRMS/MS fragmentation spectra of enzymatically formed (in vitro) and in planta NHP-OGlc and interpreted the fragments (Figure 6f, g). The spectra show dominant fragments for m/z 146.081 [M-Glc+H2O+H]+, m/z 128.070 [M-Glc+H]+ and m/z 100.075 [M-Glc+H2O-CO2+H]+. As analytical fragment for O glycosylation we present the low abundant but highly specific fragment of m/z 262.127 which represents [M-CO2+H]+. The loss of the carboxy group while the glucose-moiety is still bound to NHP is indicative for the O-glycosylation. The fragment of m/z 262.127 should not occur in case of the glucose ester. We think, that the interpretation of the LC-HRMS/MS fragments ensures the unequivocally identification of the NHP-O-glycosylation in NHP-OGlc (page 7).

We further cross-refer to the manuscripts of Bauer et al. 2020b and Holmes et al., 2020 in the discussion section. In these manuscripts the authors provide further evidence for the site of glycosylation in NHP-OGlc (page 11).

Point 3. Line 162: This is a minor point but not sure Km is always an accurate measure of substrate affinity, and so suggest modify this sentence to say simply that the enzyme has a x-lower Km for NHP/SA vs ILA. Also I did not see where the authors reported to Km for ILA in the text. Or is this statement based on Maksym et al paper? If so, I don't put much stead in Km comparisons from the different studies. Suggest to rephrase or make the ILA Km measurements themselves (which does not seem like a priority unless authors want more evidence that ILA is of minor importance).

RESPONSE: Yes, the Km-value for ILA is based on the Maksym et al paper and we fully agree with you. We rephrased the statement in accordance with your suggestion and removed the direct comparison of the Km-values for NHP and ILA in the results section (page 7). However, our Km-value for SA was in the same order of magnitude as the one in the Maksym et al paper, which suggests that one can to a certain level compare the data from this study with our data. Therefore we prefer to keep the comparison in the discussion (page 10).

Point 4. This is another minor point but some clarification would help. Based on data in Fig 6, it looks like there is as much labeled NHP in systemic leaves as in local (infiltrated) leaves. This is a bit surprising because typically only a small fraction of labeled compound would be recoverable in systemic leaves. Can the authors comment on the approx. % applied NHP that was recovered in the systemic leaves?

RESPONSE: Thank you for this valuable comment. In the local wild type leaves, we found signal intensities of about 3x105 relative peak area for D9-NHP, while we detected about 1.5x105 relative peak area in systemic wild type leaves, which is half the signal intensity detected in local leaves. The amount of D9-NHP in local leaves seems to be strongly depleted due to its efficient conversion to D9-NHP-OGlc (3.5x105 relative signal area). This conversion seems not to take place in the systemic leaves as indicated by only minor levels of D9-NHP-OGlc (1.2 x104 relative signal area) compared to the local tissue. Therefore, the amount of D9-NHP in the systemic leaves stays relatively high in comparison to the local leaves. Samples of local and systemic leaves of our tracking experiment were harvested 24 hours post infection, a time point where the response in systemic leaves just starts to establish. We assume that this is the reason for the relatively low glycosylation rate of D9-NHP observed in the systemic tissue.

The LC-HRMS analyses done for tracking the translocation of D9-NHP represent relative intensities only, because D9-NHP could not be used as internal standard for absolute quantification in this experiment. We are therefore unfortunately not able to provide data on the approx. % applied NHP that was recovered in the systemic leaves, because we can´t estimate the matrix effect. For those reasons we prefer to keep the text at it is.

Point 5. Line 231: The authors conclusion here that NHP is a "mobile signal" should be toned down because the data presented show only that exogenous (labeled) NHP CAN move, and not that endogenous NHP DOES move (many exogenous compounds, plant- and non-plant-derived, can move). The data presented are necessary but not sufficient to establish NHP as a mobile signal, in my opinion.

RESPONSE: We thank the reviewer for this important comment. In the revised version, we toned down our conclusion about NHP (page 11). Nevertheless, the ratios of D9-NHP-OGlc and D9-NHP differ a lot in local and in systemic leaves. It let us suggest, that D9-NHP-OGlc cannot be considered as the mobile signal.

Point 6. After reading the paper I came away wondering about the relative contribution/importance of SA versus NHP in SAR, and plant immunity in general. Do the new results reported here provide any insight into this question?

RESPONSE: SA and NHP seem to exhibit a synergistic role in establishing a robust answer against the attack of biotrophic pathogens. Systemic acquired resistance however might be regulated via the NHP branch. Nevertheless, to fulfill a robust defense response the plant requires functional SA and NHP metabolism and signaling. The presented results underline that increased levels of NHP and SA together result in a resistant phenotype. The data further support the notion that NHP is the signal for the establishment of systemic acquired resistance upon pathogen attack. However, from our data alone one may not conclude this directly. In the discussion, we now refer to the manuscript by the Schäffner lab (page 10), in which they show data of a NahG sid2 and NahG sid2 ugt76b1 SA deficient mutant, in addition to the ugt76b1 fmo1 double mutant. They conclude that NHP alone is not sufficient to enhance the defense reaction cassette alone, as the SA-deficient mutant is very susceptible. Altogether, a sufficient SAR response seems to require both NHP and SA.

Point 7. The title phrasing seems a bit awkward and also is not 100% accurate because the UGT negatively regulates (rather than "critical for") plant immunity. 3 suggestions below:

-The glycosyltransferase UGT76B1 modulates plant immunity through homeostatic control of N-hydroxy-pipecolic acid

-A critical role for glycosyltransferase UGT76B1 in homeostasis of N-hydroxy-pipecolic acid and plant immunity

-Modulation of N-hydroxy-pipecolic acid homeostasis and plant immunity by glycosyltransferase UGT76B1

RESPONSE: We are grateful for your suggestions and rephrased the title accordingly to: Modulation of N-hydroxy-pipecolic acid homeostasis and plant immunity by glycosyltransferase UGT76B1

Reviewer #3:

In this Mohnike et al. manuscript, the authors performed unbiased metabolome study of WT and the mutant of the UDP-glycosyltransferase, UGT76B1, and found that besides its known effect on glycosylation of the immune signal salicylic acid, the modification on another immune signal, N-hydroxy-pipecolic acid (NHP) was also compromised. Based on this initial discovery, the authors performed a series of biochemical and genetic analysis of the UGT76B1 enzyme to demonstrate its role in controlling immune activities of SA and NHP and in balancing plants' growth and defense activities.

The strength of study is the biochemical data which show a clear reciprocal relationship between the substrates and the products in the WT and the ugt76b1 mutant. However, there a few places where the logic reasoning is either unclear or not well supported:.

Point 1. Lines 72-74: "Searching for the protein that catalyzes the formation of NHP-OGlc, we found UGT76B1 as a recurring candidate gene in several studies (von Saint Paul et al., 2011; Noutoshi et al., 2012; Gruner et al., 2013; Hartmann et al., 2018)." Based on this statement, it is unclear how the authors narrowed down the gene of interest to UGT76B1 among the > 100 UDP-dependent glycosyltransferase genes in the Arabidopsis genome.

RESPONSE: We appreciate this point and explain now in more detail on page 4 why we decided to further analyze UGT76B1 as a candidate that catalyzes the formation of NHP-OGlc.

Point 2. Lines 132-148: Since transcript levels are often delinked from that of the proteins, the interpretation of mRNA measurements might not be as straightforward as presented. The significant changes in mRNA levels observed in the ugt76b1 mutant suggest that the mutation has pleiotropic effects on transcriptional activities of related genes through feedback or feedforward mechanisms which are mostly likely carried out by proteins. Therefore, the qRT-PCR data are not strong support for the statement: "Increased accumulation of NHP in ugt76b1 plants underlines the importance of turnover via UGT76B1". Short of measuring the levels of NHP synthesis enzymes, the data that support the statement appear to be in Figure 6 in which D9-NHP was infiltrated into the plants and there was a

reciprocal relationship between the level of D9-NHP and D9-NHP-Glc in WT and in ugt76b1. Since the labeled substrate was transiently introduced into plants, it reduced the pleiotropic effect in the mutant.

RESPONSE: Thank you for addressing this point. Following your suggestion, we specified the conclusion of the paragraph towards the transcript levels and toned down our statement at the end of the paragraph (page 6).

Point 3. However, the deuterium-labeling data mentioned above can only show that both D9-NHP and D9-NHP-Glc can move systemically. They cannot be used to show whether their biological activities occur locally or systemically or both. Having the data right before the SAR experiment in Figure 7 may give the reader the wrong impression that they represent the endogenous substrate and product levels during SAR. This leads to my major question for the manuscript which is on the interpretation of the infection experiment results: Are the effects of the ugt76b1 mutation observed in local infection (Figure 3) and systemic acquired resistance (Figure 7) caused by SA and/or NHP in local tissue or in systemic tissue or both? In the discussion, the authors suggest that it might be due to both. But how do we explain the complete rescue of the ugt76b1 phenotype in the ugt76b1 fmo1 double mutant and the complete lack of SAR in the fmo1 mutant? Does SA play a role in local and systemic acquired resistance? The authors need to explain the observations better by taking into consideration of the interplay between NHP, SA and ILA.

RESPONSE: Thank you for bringing up this important point. The observed enhanced resistance of ugt76b1 can most likely be explained by a feed-forward loop via the accumulation of both SA and NHP. This synergistic behavior exists in both local and systemic tissue in the same robust manner upon infection. This assumption is well supported by the data presented by Bauer and colleagues for the infection of SA-deficient mutant crossed with ugt76b1. We refer now on this manuscript (page 10). Furthermore, we included a statement about the putative influence of ILA on SA and NHP amplification in vivo (same paragraph).

In terms of SAR and especially in systemic signaling, NHP seems to play a major role, as the fmo1 1 mutant lacks a sufficient SAR response. Methyl salicylate (MeSA) and mobile SA molecules seem not to be sufficient to induce SAR without support of FMO1 and therefore NHP. NHP-caused reduction in growth can be observed in the FMO1-3D phenotype exhibiting dwarfism and significant NHP accumulation. The FMO1-3D resistant phenotype might be a result of an enhanced NHP/SA amplification cycle. In a recent publication by the Zeier lab (Schnake et al. JXB 2020), the authors discuss the individual influence of NHP and FMO1 on growth, too. When the dwarf auto-immune lines Calcium-dependent protein kinase 5 (CPK5) and Calmodulin-binding transcription activator 3 (CAMTA3) were crossed with fmo1 this mutation restores the growth deficient phenotype, without the need of an additional SA biosynthesis mutation.

Future research will have to shed light on the dissection of NHP and SAs individual downstream processes and interacting partners as for instance NHP signal perception and proliferation remains unknown. We hope that you agree that this important differentiation is beyond the scope of this work.

TPC2020-RA-00514R1 2nd Editorial decision – *accept with minor revision* November 12, 2020

We have received reviews of your manuscript entitled "Modulation of N-hydroxy-pipecolic acid homeostasis and plant immunity by glycosyltransferase UGT76B1." On the basis of the advice received, the board of reviewing editors would like to accept your manuscript for publication in The Plant Cell. This acceptance is contingent on revision based on the comments of our reviewers. In particular, please address the following:

Reviewer 1 pointed out one important concern, regarding the enzyme kinetics for the UGT76B1 glycosyltransferase, remains unresolved. As you will read in the comments below, the concern is that the authors used a biochemically invalid approach to determine kinetic values. After post-review consultation with the reviewers, I suggest a few possible options for how you and your co-authors might address this:

1. If you feel that your method for calculating Km is correct, and that your response to Reviewer 1 was unclear, you could provide additional clarification. Reviewer 1 offered the following comment in post-review discussion: "If the data to determine Km by regression analysis was acquired, it should be possible to calculate Vmax. From the Vmax value, the calculation of Kcat only requires knowledge of the initial enzyme concentration".

2. If the calculation of kinetic data remains unresolved, you can remove the kinetic analysis and modify the claim. The

reviewers agree that the kinetic data are not essential because other biochemical and genetic other data make a strong case that UGT76B1 carries out this reaction in vivo.

3. You could collect additional data to support the claim.

Please don't hesitate to contact us to discuss these options or if you have another idea for resolving this issue.

TPC2020-RA-000514R2 2nd Revision received November 17, 2020

Reviewer comments and **author responses**:

Reviewer #1:

I appreciate the additional analytical method validation data provided by the authors.

Point 1. However, I do not understand how they could have calculated Km values with end point assays and no ability to quantitatively assess conversion rates. Both Km and Kcat values are determined by varying substrate concentrations, staying within the linear range of the enzyme activity, and determining conversion rates. That is why I asked to perform the calculation for Kcat, since Km appeared to have been determined already. However, based on the response by the authors, it appears that the Km values in tables were just rough estimates based on determinations of specific activity (although even those were not quantitative). I think that the comparison of enzyme specificity (ideally by determining catalytic efficiency) is important in the context of the results presented here.

> **RESPONSE: We appreciate this advice and provide now the missing parameters via absolute quantification of the products formed by UGT76B1, which we mention now in the text and in a revised Figure 6.**

Reviewer #2:

The authors have satisfactorily my previous concerns. The revised manuscript is much improved. This work makes an important contribution to the field of plant immunity and will be appreciated by a wide audience.

Reviewer #3:

The authors have addressed my questions satisfactorily.

TPC2020-RA-00514R2 3rd Editorial decision – *acceptance pending* December 10, 2020

We are pleased to inform you that your paper entitled "Modulation of N-hydroxy-pipecolic acid homeostasis and plant immunity by glycosyltransferase UGT76B1" has been accepted for publication in The Plant Cell, pending a final minor editorial review by journal staff. At this stage, your manuscript will be evaluated by a Science Editor with respect to its presentation of scientific content, compliance with journal policies, and presentation for a broad readership.

Final acceptance from Science Editor December 21, 2020
